# Milnacipran and Vanillin Alleviate Fibromyalgia-Associated Depression in Reserpine-Induced Rat Model: Role of Wnt/β-Catenin Signaling

**DOI:** 10.1007/s12035-025-04723-w

**Published:** 2025-02-10

**Authors:** Nour A. Kamaly, Ahmed S. Kamel, Nermin Abdelhamid Sadik, Nancy N. Shahin

**Affiliations:** 1 Department of Biochemistry, Faculty of Pharmacy, Cairo University, Cairo, 11562 Egypt; 2 Department of Pharmacology and Toxicology, Faculty of Pharmacy, Cairo University, Cairo, 11562 Egypt; 3 Department of Pharmacology and Toxicology, Faculty of Pharmacy and Drug Technology, Egyptian Chinese University, Cairo, 11786 Egypt

**Keywords:** Fibromyalgia-associated depression, Wnt/beta-catenin signaling, Vanillin, Milnacipran, MicroRNAs, Nociception

## Abstract

Fibromyalgia (FM) patients are highly susceptible to depression. Wnt/β-catenin signaling has shown a crucial role against depression in several studies. The FDA-approved FM drug, milnacipran (Miln), has shown antinociceptive potential against FM. Yet, no study has investigated its antidepressant potential in FM. Vanillin (Van), a well-known phytochemical often employed as flavoring agent, has been previously reported for its antidepressant and antinociceptive effects in several animal models, but has not been tested so far in FM. This study explored the antidepressant effect of Van and Miln in FM through investigating Wnt/β-catenin signaling. FM was induced in female Wistar rats by injecting reserpine (1 mg/kg/day s.c) for 3 days. Thereafter, animals received either Miln (30 mg/kg/day p.o) or Van (100 mg/kg/day p.o) for the subsequent 14 days. Results showed that both drugs demonstrated antidepressant effect in forced swimming test besides analgesic, and antiallodynic influences observed in Randall-Selitto, hot plate, cold allodynia, Von-Frey, and tail immersion tests. Biochemically, Miln and Van significantly enhanced serotonergic transmission in the hippocampus and upregulated the protein expression of the Wnt/GSK-3β/β-catenin signaling axis, including the downstream proteins, T cell factor, and dicer. This is followed by subsequent upregulation of the resilience micro ribonucleic acids (miRNAs) 124 and 135. Histopathological examinations corroborated the biochemical and molecular findings. Interestingly, these effects of Miln and Van were overturned via administration of the β-catenin inhibitor, XAV939 (0.1 mg/kg, i.p., daily). In conclusion, this study outlined the antidepressant aptitude of Miln and Van through activating Wnt/β-catenin signaling in the hippocampus in reserpine-induced FM.

**Supplementary Information:**

The online version contains supplementary material available at 10.1007/s12035-025-04723-w.

## Introduction

Fibromyalgia (FM) presents as a chronic musculoskeletal pain condition marked by widespread and enduring pain persisting for over 3 months, with no discernible organic damage [1]. FM is usually accompanied by fatigue, poor sleep, and mood problems [1, 2]. Depression is prominent in FM with a prevalence currently amounting to 43.0% [3]. One in four FM patients is reported to have concurrent major depressive disorder, with over half experiencing major depressive disorder at some stage in their lives. The risk of encountering depressive symptoms at least once is estimated to be around 90% [4–6]. A bidirectional relationship has been found between depression and FM [7]. Moreover, studies indicate that depression has an adverse impact on the prognosis of individuals with FM, with depressed FM patients experiencing more intense pain compared to their non-depressed counterparts [5, 8]. Several studies have speculated that there may be a common pathophysiology between depression and FM [7].

The estimated prevalence of FM has been accounted to reach 2–8% of the world population [2, 9]. Interestingly, females are approximately 20 times more susceptible to FM than males [10]. This female predominance may be attributed to higher levels of anxiety and depression in women compared to men as well as the altered hormonal effects related to the menstrual cycle [11]. The pathogenesis of FM is thought to involve a combination of genetic and environmental influences.

The exact pathogenesis of FM has yet to be elucidated. However, several hypotheses have been suggested [7, 12]. The prevailing theory considers FM as a condition resulting from an amplification of pain by the central nervous system, leading to the term of nociplastic pain. Nociplastic pain refers to pain that occurs due to altered nociception in the absence of clear indications of actual or imminent tissue damage, resulting in the activation of peripheral nociceptors, without evidence of disease or injury to the somatosensory system directly causing the pain [13]. This central augmentation of pain signals, better known as central sensitization, may result from malfunctions in the pain pathways and an imbalance in brain chemicals such as serotonin and norepinephrine [2]. Notably, central sensitization was found to be associated with depressive symptoms in chronic pain diseases [12, 14]. The profound disabling impact of the disease on the patients’ quality of life results in a considerable financial burden on healthcare systems [8, 15]. So far, there is no remedy for FM and the treatment is symptomatic and multidisciplinary. Yet, the therapeutic outcomes of currently available treatments are not satisfactory enough and further research is still required for developing more effective therapeutic alternatives that can tackle all the aspects associated with the disease [12, 16].

β-Catenin, a significant component of the canonical Wnt signaling cascade, is a key molecule in chronic stress and depression [17, 18]. In experimental studies, mice lacking the β-catenin gene exhibit increased vulnerability to chronic stress induced by social defeat due to loss of important stress resilient miRNAs [17, 19, 20]. Also, the downregulation of β-catenin expression enhances stress-like behavior, whereas Wnt pathway upregulation alleviates it [21, 22]. Besides β-catenin, the biogenic amine theory stated that the depletion of biogenic amines at synaptic sites contributes to depression pathophysiology [23, 24]. There is a strong suggestion that serotonin (5-HT) and β-catenin may interact in the brain via 5-HT receptors [25, 26]. Specifically, in vivo treatment with 5-HT_1A_ receptor agonists was reported to increase glycogen synthase kinase-3 beta (GSK-3β) phosphorylated levels in the striatum, hippocampus, and prefrontal cortex [27, 28], which can in turn elevate β-catenin level [17, 29, 30]. This may outline the importance of therapeutic intervention that affects serotonergic and Wnt/β-catenin signaling in treating depression associated with FM.

Milnacipran (Miln) is a commonly used FDA-approved treatment for FM [12]. Miln functions as a selective serotonin (5-HT) and norepinephrine (NE) reuptake inhibitor [16]. It has demonstrated analgesic activity by compensating for deficits in the noradrenergic and serotonergic descending pain inhibitory pathways, as evidenced in FM and various rodent models [31–33]. The rationale for selecting Miln over other FDA-approved drugs for FM, such as duloxetine or pregabalin, lies in its clinical relevance, safety profile, and potential to offer further insights into its underlying mechanisms of action. This study aims to investigate the underlying signaling mechanisms of Miln’s antidepressant effects, which are considered less elusive compared to other FDA-approved FM treatments, such as duloxetine [34, 35]. Clinical trials have shown that Miln provides rapid symptom relief within the first week of treatment, with sustained efficacy in FM patients [35–38]. Clinical trials have evaluated Miln’s effects on a wide range of FM symptoms, including pain, mood, sleep, and cognitive function [36, 37, 39]. Notably, Miln exhibited a favorable safety and tolerability profile over duloxetine in alleviating fatigue and without the neurocognitive impairments noted with pregabalin, supporting its suitability for long-term management of FM [35, 40, 41]. Furthermore, Miln also has minimal impact on cytochrome P450 enzymes, allowing for safer combination with other medications—an important advantage in managing the complex nature of FM [34, 35, 42].

Nowadays, there has been a global shift towards drugs of herbal or natural origin due to their efficacy, affordability, and relative safety compared to synthetic drugs. Vanillin (4-hydroxy-3-methoxybenzylaldehyde, Van) is the main component of the extract of the vanilla bean (*Vanilla planifolia*, Family Orchidaceae) [43]. Van has been reported to exert several biological activities, including antidepressant [44] and antinociceptive effects [45–47]. The antidepressant effect of Van in rodents has been attributed to its antioxidant and 5-HT agonistic actions [44, 48]. However, there is a gap in elucidating the antidepressant and analgesic potential of Van in FM as well as the antidepressant effect of Miln against depression associated with FM. In addition, there is a need to explore the possible involvement of the Wnt/β-catenin signaling pathway in their serotonergic effect. Accordingly, this study aimed to evaluate the potential ameliorative effects of Miln and Van on depression associated with FM by targeting the Wnt/β-catenin signaling pathway. To confirm whether the observed effects of both drugs were mediated through this pathway, the study utilized XAV939 as a specific inhibitor [49, 50].

## Materials and Methods

### Animals

Seventy female Wistar rats, weighing 200 ± 20 g, were utilized in the present investigation. These rats were selected after a 1-day pre-screening process to exclude those with heightened sensitivity and motor abnormalities. At this phase, the study conducted the von Frey, Randall-Sellito, cold plate, and rotarod tests to assess their pain sensitivity and motor performance. These rats were procured from the National Research Centre (NRC, Giza, Egypt) and were allowed a 1-week period for acclimatization in the animal facility at the Faculty of Pharmacy, Cairo University, prior any experimental procedures. During the acclimatization and study periods, the rats were housed in groups of five per cage in standard polycarbonate cages with dimensions of 40 cm × 25 cm × 15 cm. The environmental conditions were tightly controlled, with a constant room temperature maintained at 25 ± 2 °C, relative humidity of 60 ± 10%, and a 12-h light/dark cycle (lights on from 7:00 AM to 7:00 PM). The lighting conditions were carefully monitored, using dim red light to facilitate observation without significantly affecting the rats’ perception of the dark phase. The cages were cleaned and bedding was replaced daily to ensure hygiene. The animals were provided ad libitum access to standard chow pellets (National Research Centre diet, Giza, Egypt) and tap water throughout the experimental duration. The room’s temperature and humidity were monitored daily to minimize any environmental fluctuations that could impact the experimental outcomes.

### Compliance with Ethical Standards

The procedures conducted in this study rigorously adhered to the guidelines outlined in the Care and Use of Laboratory Animals Guide (NIH publication No. 85–23, revised 2011). The experimental protocol underwent review and approval by the Research Ethics Committee at the Faculty of Pharmacy, Cairo University (permit number: BC (2706)). Following the acclimatization period, the animals underwent motor and nociplastic behavioral tests, with a 2-h resting interval between each pair of tests. It was ensured that the test sequence started with exploratory activity and concluded with the most stressful task. Behavioral assessments were conducted during the light phase in a sound-insulated setting, with testing arenas being regularly sterilized with 70% ethanol following every test. The utmost efforts were exerted to minimize distress to the animals during the investigation.

### Drugs and Chemicals

Reserpine (Res) (Cat # R0875), vanillin (Van) (Cat # W310727), and XAV939 (2-[4-(trifluoromethyl) phenyl]−1,5,7,8-tetrahydrothiopyrano[4,3-d] pyrimidin-4-one) (Cat # X3004) were acquired from Sigma-Aldrich Chemical Co. (St. Louis, MO, USA). Milnacipran hydrochloride (Miln) (Myodonia®) was obtained from Amoun Pharmaceutical Co. SAE (Egypt). Glacial acetic acid (Cat # A6283, Sigma-Aldrich Chemical Co. (St. Louis, MO, USA)) was used to dissolve Res and was diluted with distilled water to a final concentration of 0.5% acetic acid [51], whereas Van and Miln were dissolved in distilled water [52, 53]. XAV939 was initially dissolved in 2% dimethyl sulfoxide (DMSO) (Merck, Germany; Cat #1,029,521,000) and subsequently diluted with distilled water to achieve the desired working concentration prior to use. Other chemicals utilized were of the highest analytical quality.

### Induction of FM

Experimental FM model was induced in female rats as was previously reported [51]. Briefly, Res (1 mg/kg) was subcutaneously (s.c.) administered every 24 h for three consecutive days. To simulate the real-life prevalence of FM among women, the study specifically utilized female rats as the model. This choice reflects the higher incidence of FM in females, who represent approximately 80–96% of cases [54, 55]. Thus, the study aimed to evaluate the therapeutic effects of milnacipran and vanillin in a Res-induced FM model, aligning with FM’s gender-specific impact [56, 57].

### Experimental Design

**Fig. 1:**
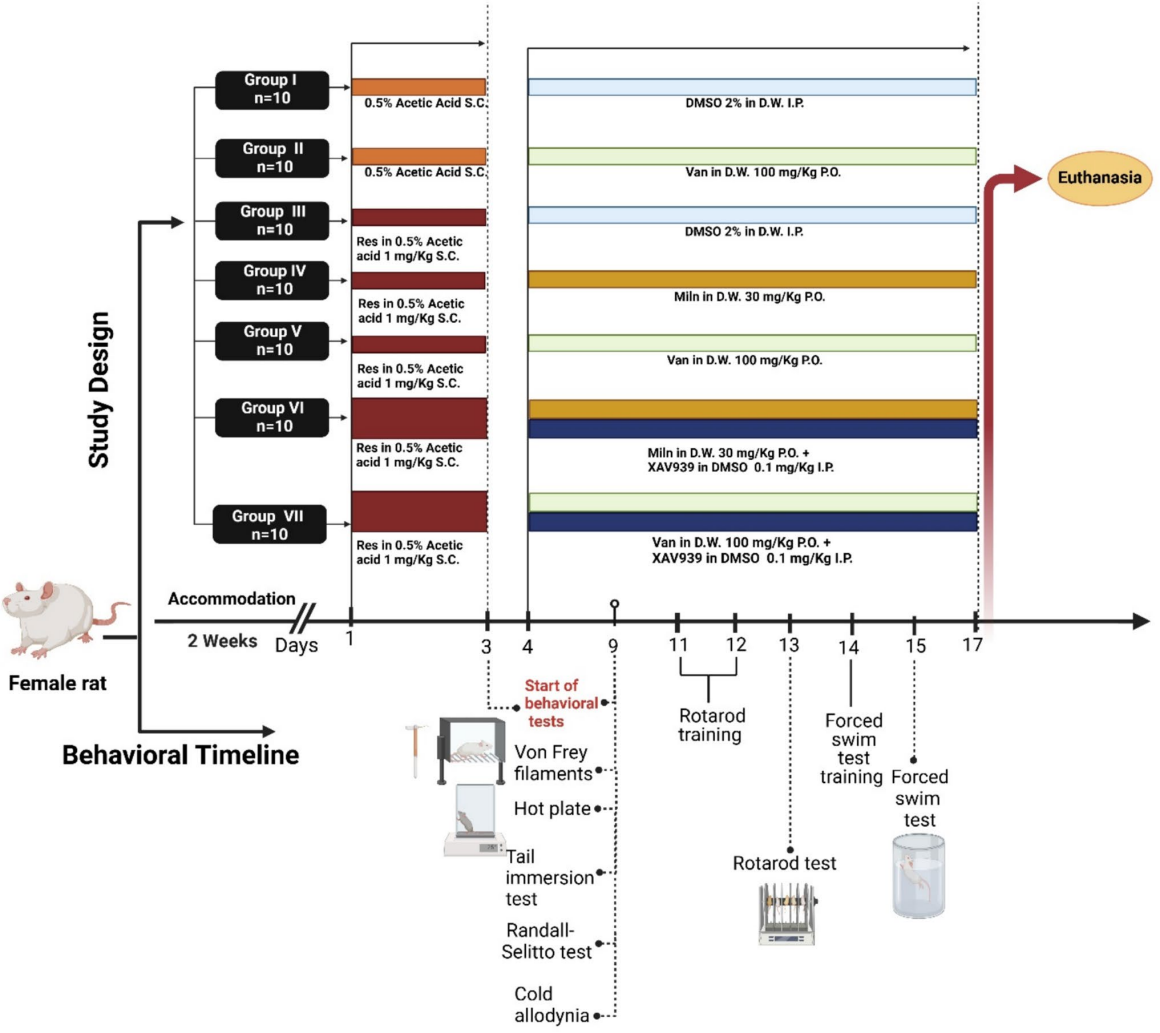
Illustration of the experimental design and the behavioral experiments’ timeline. Res, reserpine; Van, vanillin; Miln, milnacipran; S.C, subcutaneous; P.O, per oral; I.P, intraperitoneal

As shown in Fig. 1, the female rats were randomly assigned to 7 groups (*n* = 10/group). The sample size was determined using prior studies as a reference and was validated using the G*Power software (version 3.1, Düsseldorf, Germany). The parameters for the power analysis were as follows: an effect size of 0.5, an alpha level of 0.05, and a power of 0.8. The groups were group I (control), group II (Van control), group III (FM), group IV (FM + Miln), group V (FM + Van), group VI (FM + Miln + XAV939), and group VII (FM + Van + XAV939).

Rats in *group I* (*control*) received a daily s.c. injection of 0.5% glacial acetic acid, the vehicle for Res, for 3 days, followed by daily oral administration of 2% DMSO in distilled water, serving as the vehicle for drugs and inhibitors, for the subsequent 14 days. *Group II* (*Van control*) received daily s.c. injections of 0.5% glacial acetic acid for 3 days, followed by oral administration of Van (100 mg/kg/day, p.o.) for the next 14 days [52]. *Group III* (*FM model*) received Res (1 mg/kg/day, s.c.) for 3 days, followed by 2% DMSO in distilled water as the vehicle, administered orally for the following 14 days. Rats in *groups IV–VII* received Res as in group III for the initial 3 days. Starting on day 4, group IV received Miln (30 mg/kg/day, p.o.) for 14 days [53], while group V received Van (100 mg/kg/day, p.o.) for the next 14 days. *Group VI* received the Wnt inhibitor XAV939 (0.1 mg/kg/day, intraperitoneally [i.p.]) 30 min prior to Miln (30 mg/kg/day, p.o.) for 14 days [58]. *Group VII* received the Wnt inhibitor XAV939 (0.1 mg/kg/day, i.p.) 30 min prior to Van (100 mg/kg/day, p.o.) for 14 days.

### Behavioral Tests

The animals were pre-screened prior to the study to assess for any motor impairments’ ineligibility to perform motor tests or heightened sensitivity. All animals included in the study met the eligibility criteria for motor performance and pain assessments. The behavioral tests conducted in the current study comprised (i) nociceptive pain tests including the following: Randall-Selitto test, hot plate test, cold plate test, and tail immersion test; (ii) rotarod test to test motor coordination; (iii) forced swim test as a test for depressive-like behavior; and (iv) Von Frey test as a test for mechanical allodynia. These tests were carried out in accordance with a fixed schedule adopted in all the rats in all groups. As illustrated in Fig. 1, the baseline of rats’ performance in nociceptive tests was recorded on day 3 after FM induction and before Miln and Van administrations. Then, the tests were reconducted on day 9 testing in the following order of least painful to most painful to minimize animal stress: Von Frey, tail immersion, cold plate, hot plate, and Randall-Selitto tests. On the 13th day, rotarod test was conducted after two previous days of training (days 11–12). Then on day 15, the forced swim test was performed after rats underwent a previous training session on day 14.

#### Randall-Selitto Test

Randall-Selitto pressure analgesiometer is a frequently employed tool for evaluating mechanical hyperalgesia by measuring the withdrawal threshold (Model 7200, Ugo Basile, Italy). The instrument consists of a blunted probe ensuring that no injury or damage is inflicted. This was accomplished by applying a steadily increasing mechanical force to the mid-gastrocnemius muscle of the rat’s hind limb. A soft cotton cloth was used to gently immobilize the rats during the measurement. The point at which the hind limb was withdrawn was considered the endpoint and the average of three consecutive tests with an inter-stimulus interval of 1 min was considered the muscle pressure threshold. To avoid any tissue damage, a maximum load of 250 g was set as the cutoff limit in this test [59].

#### Hot Plate Test

The hot plate test is employed to measure thermal hyperalgesia in animals. The rats were positioned on an enclosed hot plate (20 cm diameter × 31 cm height) that is non-escapable (Model 7280, Ugo Basile, Italy). The temperature was kept at 55 °C ± 1. Each rat was observed, and the end point was established to be the licking of a hind paw or jumping out of the apparatus. A maximal cutoff was decided at 12 s to minimize tissue damage [60].

#### Hind Paw Cold Allodynia Test

The pain sensitivity of the animals was evaluated by submerging each rat’s hind paw in a cold-water stimulus (4.5 °C) and measuring the hind paw withdrawal latency (HPWL). One immersion was confined to one hind paw at a time to limit tissue injury. The test was carried out two times on each hind paw with a 5-min break between both trials, then the HPWL was computed by averaging the values obtained from both hind paws for every rat. The relationship between HPWL and allodynia is inversely proportional [61].

#### Tail Immersion Test

The tail immersion test is utilized to evaluate spinal thermal sensitivity in rats. The distal 1 cm part of the tail was submerged in a water bath kept at a consistent temperature of 55 °C and the time taken for a nociceptive reaction, measured in seconds, was recorded. The nociceptive response was either tail withdrawal, a forceful tail shaking, or a quick reflexive movement of the entire body of the animal. To avoid tissue damage, a maximum time limit of 15 s was set [60].

#### Rotarod Test

The rotarod task is utilized to evaluate the motor coordination and balance of rodents. Prior to the start of the study, all animals were pre-screened on the rotarod for 1 day to assess their motor abilities and ensure they could successfully perform the task. All animals that stayed on the rotating rod for over 1 min were included in the subsequent analyses. The rats underwent a 2-day training period, involving three trials per day, on an automated device consisting of five lanes (Model 47,750, UgoBasile, Italy) rotating at a speed accelerating from 4 to 40 rpm with a 3-min cut-off time. On the day of testing, three trials were conducted for each rat wherein it was placed on the rotating rod for 3 min per trial and the average rotarod fall-off latency (RRFOL) for each rat was determined [62, 63].

#### Forced Swimming Test

The forced swimming test is used for the assessment of depressive behavior in rats and mice [64]. Each rat was placed in a plastic cylinder with the dimensions of 20 cm (diameter) by 50 cm (height) that was filled to a depth of 30 cm with water maintained at 23–25 °C. This ensured that the rats were not able to touch the cylinder’s base with their paws or tails for support. The rats underwent a training session 1 day prior to the testing day. A standard 5-min test period was allocated for each rat during which the total immobility time is recorded using a stopwatch. Immobility time was defined as when the rat stops swimming and just floats on the water surface without exerting any effort other than to keep its head above water. A heating lamp was used to keep the animals warm and avoid hypothermia [65].

#### Von Frey Test

Mechanical allodynia is evaluated in rodents via the Von Frey filament test. Rats were put individually in small cages with mesh bottoms and were allowed to acclimate to the testing environment for 15 min. Von Frey filaments (Model 37,450–275, Ugo Basile, Italy) with calibrated bending forces, measured in grams, of different intensities were employed to give punctuate mechanical stimuli of different intensities. The process involved applying the monofilament from beneath the mesh floor to the plantar surface of the hind-paw of the rat with enough force to induce slight bending and holding it for 1 s beginning with the least filament force. Each stimulation was repeated five times, with an interval of 4–5 s between stimuli. The withdrawal threshold, defined as the force in grams at which the rat removed its paw, was recorded [60].

### Sampling

At the conclusion of the experimental period, following the completion of behavioral tests, rat euthanasia was achieved via decapitation under light thiopental anesthesia at a dosage of 40 μg/kg [66, 67]. Subsequently, whole brains were harvested, rinsed with ice-cold isotonic saline, and dried. The harvested whole brains from each group were divided into two subsets. One subset (*n* = 3 per group) was promptly fixed in 10% formalin/saline for histopathological examination. In the second subset (*n* = 7 per group), hippocampi were swiftly dissected out. The hippocampi from one hemisphere of the brain were homogenized in phosphate-buffered saline (PBS) for use in enzyme-linked immunosorbent assays (ELISA) (*n* = 6), while the hippocampi from the other hemisphere underwent processing for quantitative reverse transcriptase polymerase chain reaction (qRT-PCR) (*n* = 3) and Western blotting analyses (*n* = 3). Throughout the sample analysis, all evaluators were unaware of the identity of the samples wherein the sample coding and decoding were performed by a separate investigator.

### Histopathological Examination

Brain tissue specimens were preserved in 10% neutral buffered formalin for 72 h. Subsequently, the formalin-fixed brain sections underwent trimming and were prepared for paraffin embedding through a series of steps including dehydration in varying concentrations of ethanol, clearing in xylene, infiltration, and embedding into Paraplast media. Sagittal brain sections, 4 μm in thickness, were then cut using a rotary microtome to visualize the hippocampal regions across different samples. The tissue sections were subjected to staining with hematoxylin and eosin (H&E) for general histopathological examination, as well as toluidine blue stain (Nissl stain) to distinguish between damaged and intact neurons. A skilled histologist, unaware of the sample assignments for the different study groups, inspected the stained tissue sections using a light microscope. Subsequently, a minimum of six randomly selected, non-overlapping fields were scanned for the CA3 and dentate gyrus (DG) regions for each sample to quantify the average counts of intact neurons in the toluidine blue-stained sections. A 4-point scoring system was employed to evaluate the severity of histopathological lesions. The scale was defined as follows: 0 = negative record, 1 = mild records in less than 15% of examined tissue sections, 2 = moderate records in 16–35% of examined tissue sections, 3 = severe records in more than 35% of examined tissue section. All examinations under the light microscope and image analyses were conducted using the Leica application module for histopathological analysis, integrated with a full HD microscopic imaging system (Leica Microsystems GmbH, Wetzlar, Germany).

### Biochemical Investigations

#### ELISA Assays

The hippocampal serotonin (5-HT) and T cell factor (TCF) levels were determined using rat-specific ELISA kits (Cat # MBS9362408 and MBS166467, respectively, Mybiosource, CA, USA) in accordance with the guidelines provided by the manufacturer with the respective kits. The method described by Bradford (1976) was used to measure the protein content in hippocampal homogenates.

#### Western Blotting Analysis

The protein levels of Wnt3a, phosphorylated and total GSK-3β, β-catenin, and dicer were assessed using Western blot technique. The hippocampi were homogenized in radioimmunoprecipitation assay (RIPA) lysis buffer (Cat # PL005, Bio BASIC INC., Ontario, Canada) and assayed for protein content using a Bradford protein assay kit (Cat # SK3041, Bio BASIC INC., Ontario, Canada). Subsequently, a 20-μg protein aliquot of each sample was mixed with an equal volume of 2 × Laemmli sample buffer (Cat # 1,610,737, Bio Rad, California, USA), separated by sodium dodecyl sulfate–polyacrylamide gel electrophoresis, and then conveyed onto a polyvinylidene difluoride membrane. Membrane blocking was achieved using 3% bovine serum albumin in tris-buffered saline with Tween 20 (TBST), followed by 24-h incubation at 4 °C with primary antibodies (1:1000 dilution) specific to β-catenin (Cat # 71–2700, Thermo Fisher Scientific, USA), Wnt3a (Cat # PA5-119,852, Thermo Fisher Scientific, USA), Dicer (Cat # PA5-115,124, Thermo Fisher Scientific, USA), total GSK-3β (Cat # PA5-29,251, Thermo Fisher Scientific, USA), and phosphorylated GSK-3β (Cat # MA5-14,873, Thermo Fisher Scientific, USA). Following three washes for 15 min each with TBST, horseradish peroxidase-conjugated secondary antibodies (Goat anti-rabbit-HRP-lmg Goat mab-Novus Biologicals, USA) were added and incubated for 1 h at room temperature. Quantitative analysis of protein band density on the blot was carried out using enhanced chemiluminescent substrate (Cat # 170–5060, ClarityTM Western ECL substrate-BIO-RAD, USA). Band intensities were assessed using Chemi Doc MP Imaging System with Image LabTM software version 5.1 (Bio-Rad Laboratories Inc., Hercules, CA, USA) and were normalized to β-actin as a reference control.

#### Gene Expression Analysis by Quantitative Real-Time Polymerase Chain Reaction

Gene expression levels of miRNA-124, miRNA-135, and serotonin receptor (5-HT_1A_) were assayed using quantitative real-time polymerase chain reaction (qRT-PCR). Total RNA was isolated from hippocampal tissue using SV total RNA isolation system (Cat # Z3100, Promega, Madison, WI, USA). The extracted RNA was utilized for the conversion to cDNA employing a high-capacity cDNA reverse transcription kit (Cat # K4374966, Thermo Fisher Scientific, USA). Real-time qPCR amplification and analysis were conducted using SYBR Green Master Mix (Cat # K0221, Thermo Fisher Scientific, MA, USA) and an Applied Biosystems software (version 3.1, StepOne™, USA). A miRNeasy Micro Kit (Cat # 217,084, QIAGEN, MD, USA) was used for miRNA extraction to determine miRNA-124 and miRNA-135 expression, followed by cDNA synthesis by means of a TaqMan microRNA reverse transcription kit (Cat # 4,366,596, QIAGEN, MD, USA). The primer sequences used in this investigation (Invitrogen Thermo Fisher Scientific, MA, USA) are displayed in Table 1. The implemented PCR protocol consisted of an initial DNA polymerase activation step at 95 °C for 10 min, succeeded by 40 cycles of denaturation at 95 °C for 15 s, and annealing/extension at 60 °C for 60 s. The target genes’ relative expression levels were computed following the 2^− ΔΔCt^ method (Schmittgen and Livak, 2008) where the internal control for serotonin receptor gene expression was β-actin, while that for miRNA-135 and miRNA-124 expression was U6.

**Table 1:** Primer sequences used in gene expression analysis

Gene	Primer sequence	Accession number
miRNA-124	Forward: 5′-CTCTCTCTCCGTGTTCACAGC-3′ Reverse: 5′-ATTCTTGGCATTCACCGCGT-3′	NR_031866
miRNA-135	Forward: 5′-GCTTATGGCTTTTCATTCCT-3′ Reverse: 5′-GTGCAGGGTCCGAGGT-3′	NR_031881
U6	Forward: 5′-CTCGCTTCGGCAGCACATATACTA-3′ Reverse: 5′-ACGAATTTGCGTGTCATCCTTGCG-3′	XR_005498700.1
Serotonin receptor (5-HT_1A_)	Forward:5′-AGAAGCCACCTTGTGTGTGA-3′ Reverse: 5′-TTGCTCATTGCTGATGGACT-3′	NM_012585.2
β-Actin	Forward:5′-CTACCTCATGAAGATCCTCACC-3′ Reverse:5′-AGTTGAAGGTAGTTTCGTGGAT-3′	NM_031144.3

### Statistical Analysis

All data in the study were assessed for normality and homogeneity of variance using the D’Agostino–Pearson and Bartlett’s test, respectively. Data that did not meet normality assumptions were analyzed using the Kruskal–Wallis nonparametric one-way ANOVA followed by Dunn’s multiple comparisons test, with results expressed as median ± range. While data that met normality assumptions but did not meet homogeneity of variance were analyzed using Welch’s ANOVA followed by Dunnett’s T3 multiple comparison test and results expressed as median ± range. Biochemical data that met both normality and homogeneity of variance assumptions were analyzed using one-way ANOVA followed by Tukey’s multiple comparisons test, with results expressed as mean ± SD. GraphPad Prism software (version 9) was employed for statistical analysis and data visualization. A significance level of *P* < 0.05 was applied for all tests. The F-value (*F*), Eta squared (η2), and statistical significance (*P*) were calculated for each effect.

## Results

### Milnacipran and Vanillin Ameliorated the Mechanical Hyperalgesia and Mechanical Allodynia Associated with Reserpine in Rats

**Fig. 2:**
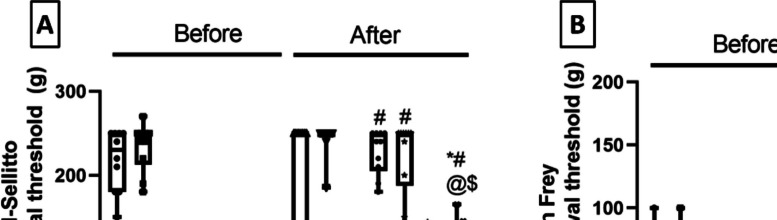
Effect of milnacipran and vanillin alone and combined with XAV939 on reserpine-induced mechanical hyperalgesia and mechanical allodynia in rats. Mechanical hyperalgesia (**A**) was examined using the Randall-Selitto apparatus and mechanical allodynia (**B**) was assessed by the Von-Frey test. The behavioral data were expressed as median ± range (*n* = 10). *vs CTRL, #vs FM, ^@^ vs FM + Miln and ^$^ vs FM + Van using Kruskal–Wallis test followed by Dunn’s multiple comparison test; *P* < 0.05. CTRL, control; VAN, vanillin; FM, fibromyalgia; Miln, milnacipran; XAV, XAV939

Mechanical hyperalgesia was reflected by limb withdrawal threshold in the Randall-Selitto apparatus (Fig. 2A). On day 3, Res-induced mechanical hyperalgesia and allodynia aberrations in all groups were compared to control rats. On day 9, rats in the FM model group suffered a significant 79.9% decrement in the limb withdrawal threshold compared to the control group. Meanwhile, the administration of Miln or Van counteracted this Res-induced anomaly by increasing the limb withdrawal threshold to 4.88- and fivefold compared to the FM group, respectively. Importantly, the use of XAV939 hampered the reinstating effects of the tested drugs on the measured thresholds resulting in values that were considerably lower than those in their XAV939-free counterparts. Miln + XAV939 rats showed a 74.4% decrement in the limb withdrawal threshold compared to their XAV939-free counterparts. Additionally, Van + XAV939 rats displayed a decrement of 42.8% in the limb withdrawal threshold compared to their XAV939-free counterparts.

On the other hand, mechanical allodynia was reflected by paw withdrawal threshold measured via the Von Frey apparatus (Fig. 2B). FM group rats displayed a marked 70% decrease in the paw withdrawal threshold compared to the control group, whereas the administration of Miln or Van counteracted this Res-associated decrement by increasing the paw withdrawal threshold to 3.5- and 4.87-fold compared to the FM group, respectively. However, the use of XAV939 reversed the increasing effect of Miln and Van on measured thresholds resulting in values that were significantly lower than those in their XAV939-free counterparts. Miln + XAV939 rats showed a 64.59% decrement in the limb withdrawal threshold compared to their XAV939-free counterparts. Additionally, Van + XAV939 rats displayed a decrement of 74.26% in the limb withdrawal threshold compared to their XAV939-free counterparts.

### Milnacipran and Vanillin Mitigated the Thermal Hyperalgesia, Cold Allodynia, and Spinal Thermal Sensitivity Associated with Reserpine in Rats

**Fig. 3:**
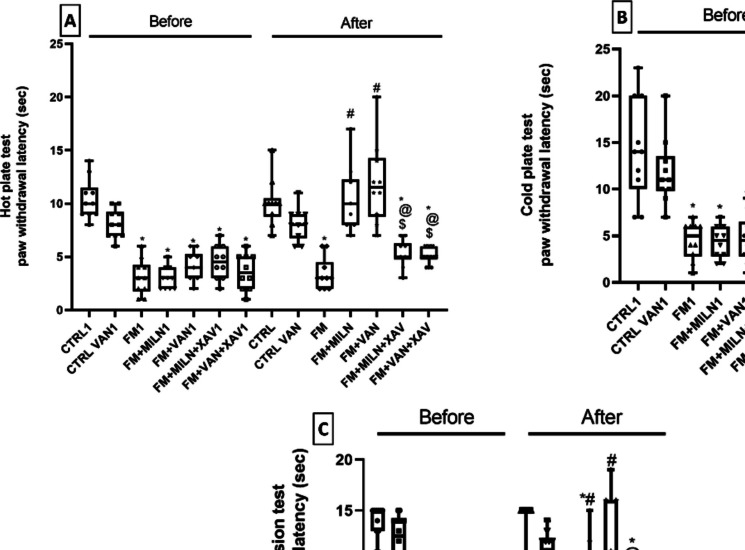
Effect of milnacipran and vanillin alone and in conjunction with XAV939 on reserpine-induced thermal hyperalgesia, cold allodynia, and spinal thermal sensitivity in rats. Thermal hyperalgesia was tested using the hot plate test (**A**), cold allodynia was assessed using the cold plate test (**B**), and spinal thermal sensitivity was examined through the tail immersion test (**C**). The behavioral data were expressed as median ± range (*n* = 10). *vs CTRL, #vs FM, ^@^ vs FM + Miln and.^$^ vs FM + Van using Welch’s ANOVA followed by Dunnett’s T3 multiple comparison test for hot plate data while Kruskal–Wallis test followed by Dunn’s multiple comparison test was used for cold plate and tail immersion tests; *P* < 0.05). CTRL. control; VAN, vanillin; FM, fibromyalgia; Miln, milnacipran; XAV, XAV939

On day 3, all groups subjected to Res showed heightened sensitivity to hot-plate, cold allodynia, and spinal thermal sensitivity compared to Res untreated rats. On day 9, thermal hyperalgesia was tested by the hot plate test (Fig. 3A) in which the FM group rats displayed a profound 66.3% decrease in the paw withdrawal latency (PWL) compared to the control group, whereas Miln and Van treatment resulted in delaying the hot plate PWL to 3- and 3.5-fold when compared to the FM group, respectively. Addition of XAV939 to Miln and Van shortened the hot plate PWL by 49% and 55.5% respectively compared to their XAV939-free counterparts.

Meanwhile, in the test for cold allodynia, cold plate test (Fig. 3B) Res-injected rats displayed a significant 72% reduction in PWL compared to the control group. Additionally, Miln and Van counteracted Res’ effect by prolonging the PWL to 3.59- and 4.11-fold, respectively, compared to the FM group. On the other hand, XAV939 administration with either Miln or Van reversed their ameliorative effect by shortening the PWL by 69.6% and 61%, respectively, compared to their XAV939-free counterparts.

Additionally, Res-injected rats suffered from increased spinal thermal sensitivity as tested by the tail immersion test (Fig. 3C) in the form of a profound 83% decrement in the tail withdrawal latency compared to the control group. Treatment with Miln or Van reversed Res’ shortening effect and prolonged the tail withdrawal latency to 3.78- and 4.45-fold, respectively, compared to the FM group. However, the coadministration of XAV939 with either Miln or Van abolished the reinstating effects of the tested drugs shortening the tail withdrawal latency by 58.4% and 40.7% compared to their XAV939-free counterparts.

### Milnacipran Improved Motor-Incoordination in Reserpinized Rats, While Milnacipran and Vanillin Ameliorated Depressive-Like Behavior Associated with Reserpine

**Fig. 4:**
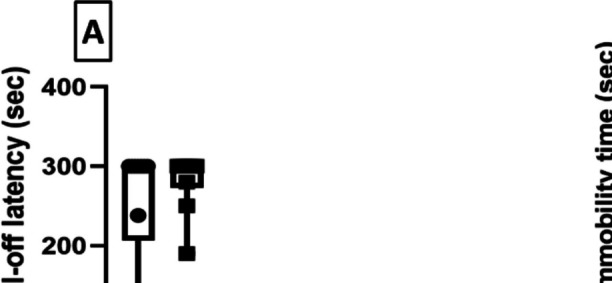
Effect of milnacipran and vanillin alone and in combination with XAV939 on reserpine-induced motor-incoordination and depression in rats. Motor-incoordination was examined via the rotarod apparatus (**A**) and depressive behavior was assessed by the forced swim test (**B**). The behavioral data were expressed as median ± range (*n* = 10). *vs CTRL, #vs FM, ^@^ vs FM + Miln and.^$^ vs FM + Van using Kruskal–Wallis test followed by Dunn’s multiple comparison test; *P* < 0.05). CTRL, control; VAN, vanillin; FM, fibromyalgia; Miln, milnacipran; XAV, XAV939

As depicted in Fig. 4A, the use of Res negatively impacted the rodents’ motor-coordination in the FM group which was reflected as a reduced rotarod fall-off latency (RRFOL) by 76.5% in comparison with the control group. Miln administration improved motor coordination in Res-treated rats by significantly increasing the RRFOL to 3.22-fold compared to the FM group. The coadministration of XAV939 with Miln attenuated its protective effects resulting in decreased RRFOL by 63.2% relative to the corresponding XAV939-free group. On the other hand, the administration of Van improved the coordination but did not mount to any significance compared to the FM group. Additionally, the Van + XAV939 group did not vary significantly from the corresponding XAV939-free group.

The Res group displayed significant depression-indicating behavior during the forced swimming test (Fig. 4B) featured by increased immobility time to reach 4.69-fold compared to the control group. Miln or Van administration to Res-challenged rats proved to have an antidepressant effect by decreasing the immobility time by 35.9 and 36.2%, respectively, compared to the FM group. XAV939 coadministration, however, abrogated the ameliorative effects of Miln and Van. This was exhibited by a marked prolongation of the immobility time to 1.86- and 1.83-fold versus their XAV939-free counterparts, respectively.

### Reserpine Reduced the Rat Hippocampal Level of Wnt3a, While Milnacipran and Vanillin Effectively Increased Its Level

**Fig. 5:**
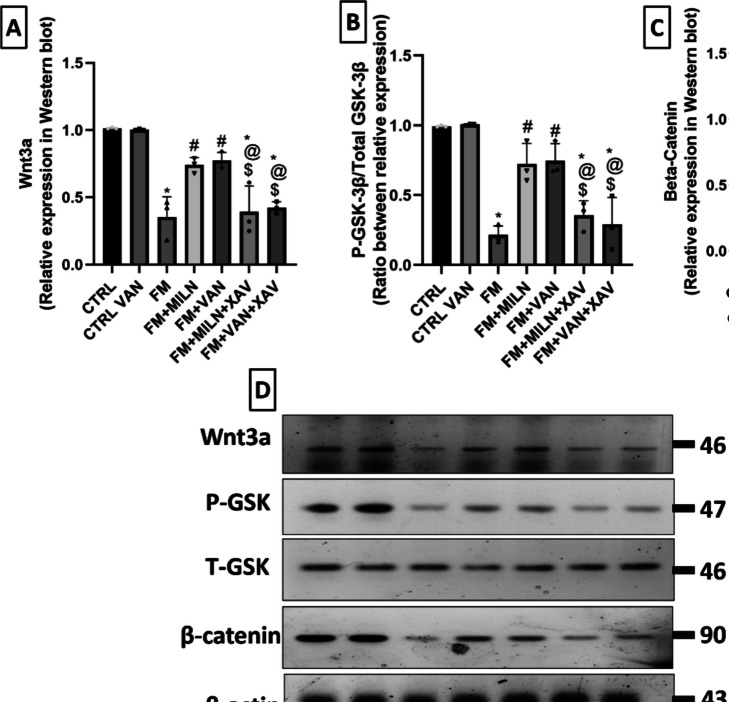
Effect of milnacipran and vanillin alone and combined with XAV939 on reserpine-induced alterations in rat hippocampal levels of **A** Wnt3a, **B** p-GSK3β/t-GSK3β ratio, and **C** beta-catenin all assessed through western blot. **D** Representative western blot images of changes in the expression level of Wnt3a, p-GSK3 β, t-GSK3 β, and β-catenin where β-actin was used as an internal control to calculate relative expression. Each bar with vertical line represents the mean ± SD (*n* = 3). *vs CTRL, #vs FM, ^@^ vs FM + Miln and ^$^ vs FM + Van using one-way ANOVA followed by Tukey’s multiple comparisons test; *P* < 0.05. CTRL, control; VAN, vanillin; FM, fibromyalgia; Miln, milnacipran; XAV, XAV939; p-GSK3β/t-GSK3β, phosphorylated to total glycogen synthase kinase 3 beta

Wnt3a showed significant difference between groups: *F*_(6, 14)_ = 24.71, *P* < 0.0001, η2 = 0.914. As shown in Fig. 5A, Res-injected rats displayed a significant 65% decrease in the level of Wnt3a in the rodent hippocampus contrasted with the control group. The introduction of Miln or Van mitigated the effect of Res increasing the Wnt3a level to 2.09- and 2.18-fold compared to the FM group, respectively. XAV939 counteracted the effects of Miln and Van on Wnt3a, where it displayed similar results to the FM group. This was exhibited in Miln + XAV939 and Van + XAV939 by 46.8% and 45.2% decrements in Wnt3a levels versus their XAV939-free counterparts, respectively.

*Reserpine Decreased the Rat Hippocampal Phosphorylated Glycogen Synthase Kinase 3 Beta Level and Accordingly the Level of Beta-Catenin, Whereas Milnacipran and Vanillin Caused Their Increase.*

The ratio between p-GSK-3β/t-GSK-3β and the β-catenin level was significantly different between the groups (for p-GSK-3β/t-GSK-3β ratio: *F*_(6, 14)_ = 25.80, *P* < 0.0001, η2 = 0.916; and for β-catenin: *F*_(6, 14)_ = 39.63, *P* < 0.0001, η2 = 0.944).The Res-induced FM group exhibited significant 78.3% decrease in the hippocampal phosphorylated to total GSK-3β ratio (Fig. 5B) compared to the control group. This in turn led to the decrease in β-catenin level (Fig. 5C) in the FM group by 66.6% when compared to the control group. However, the concomitant administration of Miln counteracted Res’ effect by raising the p-GSK-3β/t-GSK-3β ratio to a 3.36-fold, and in turn the β-catenin level to a 2.32-fold compared to the FM group. Similarly, Van administration counteracted Res’ effect by raising the p-GSK-3β/t-GSK-3β ratio to a 4.47-fold, and in turn the β-catenin level to a 2.3-fold compared to the FM group.

It is worthy to note that the β-catenin inhibitor, XAV939, averted the restorative effect of Miln and Van on the p-GSK-3β/t-GSK-3β ratio and the β-catenin level. In XAV939 + Miln rats, there was a significant 50.9% decrement in the ratio of p-GSK-3β to t-GSK-3β versus their XAV939-free counterparts. This was reflected in the β-catenin level, as well, which decreased in Miln + XAV939 rats by 48.5% compared to their XAV939-free counterparts. Similarly, in Van + XAV939 rats, there was a significant 61.1% decrease in the p-GSK-3β/t-GSK-3β ratio relative to the XAV939-free Van group. This was also reflected as a profound decrease in β-catenin level in Van + XAV939 rats by 48% when compared with their XAV939-free counterparts.

### Reserpine Had a Diminishing Effect on The Rat Hippocampal TCF and Dicer Levels, Whereas Milnacipran and Vanillin Had an Augmenting Effect on Both in Rats

**Fig. 6:**
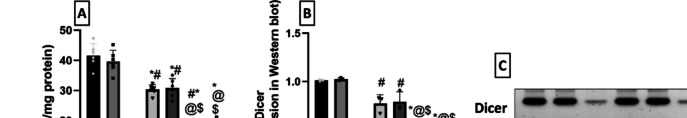
Effect of milnacipran and vanillin alone and combined with XAV939 on reserpine-induced alterations in rat hippocampal levels of **A** TCF measured by ELISA and **B** dicer enzyme measured by Western blot. **C** Representative Western blot images of changes in the expression level of dicer where β-actin was used as an internal control to calculate relative expression. Each bar with vertical line represents the mean ± SD (for TCF n = 6, for Dicer n = 3). *vs CTRL, #vs FM, ^@^ vs FM + Miln and ^$^ vs FM + Van using one-way ANOVA followed by Tukey’s multiple comparisons test; *P* < 0.05. CTRL, control; VAN, vanillin; FM, fibromyalgia; Miln, milnacipran; XAV, XAV939; TCF, T cell factor

TCF and dicer levels were significantly different between the groups (for TCF: *F*_(6, 35)_ = 96.49, *P* < 0.0001, η2 = 0.942; and for dicer: *F*_(6, 14)_ = 40.87, *P* < 0.0001, η2 = 0.946). Res-injected rats showed significant 69.2% reduction in the TCF (Fig. 6A) hippocampal level in contrast with the control group. On the contrary, treatment with Miln and Van led to 2.36- and 2.4-fold elevations in TCF compared to the FM group, respectively. The co-administration of XAV939 deterred the effects of Miln and Van on TCF levels. Specifically, Miln + XAV939 and Van + XAV939 rats displayed 39.4% and 42.8% lower TCF levels than their XAV939-free counterparts, respectively.

Additionally, dicer hippocampal levels were decreased significantly by 76.2% in the FM group compared to the control group (Fig. 6B). This was mitigated by Miln and Van treatment increasing the dicer level to 3.22- and 3.29-fold compared to the FM group, respectively. Furthermore, the addition of XAV939 to Miln and Van hampered their increasing effect. This was exhibited by a 47.4% and 50.2% decrease in dicer level in Miln + XAV939 and Van + XAV939 respectively compared to their XAV939-free counterparts.

### Reserpine Downregulated the Rat Hippocampal Expression of Stress-Resilient miRNAs miR-124 and miR-135, While Milnacipran and Vanillin Boosted Their Expression

**Fig. 7:**
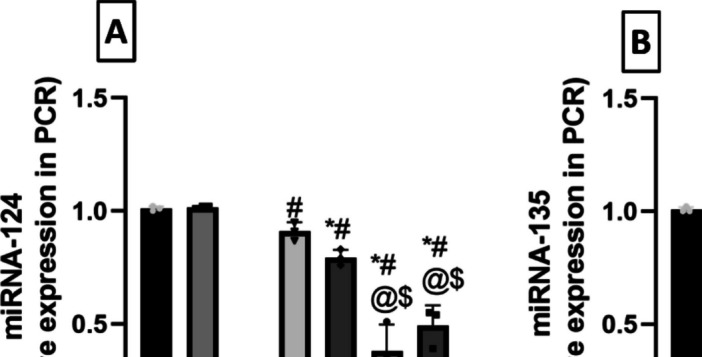
Effect of milnacipran and vanillin alone and in combination with XAV939 on reserpine-induced alterations in rat hippocampal relative miRNA expression of **A** miRNA-124 and **B** miRNA-135 determined by qRT-PCR. Each bar with vertical line represents the mean ± SD (*n* = 3). *vs CTRL, #vs FM, ^@^ vs FM + Miln and.^$^ vs FM + Van using one-way ANOVA followed by Tukey’s multiple comparisons test; *P* < 0.05. CTRL, control; VAN, vanillin; FM, fibromyalgia; Miln, milnacipran; XAV, XAV939

The level of miRNA-124 varied significantly between the groups (*F*_(6, 14)_ = 89.34, *P* < 0.0001, η2 = 0.974). FM rats displayed a significant 81.8% reduction in miRNA-124 compared to the control group (Fig. 7A), whereas Miln and Van treatment abolished this Res-induced decrease by increasing miRNA-124 to 4.96- and 4.32-fold compared to the FM group. The addition of XAV939 significantly attenuated the restorative effects of Miln and Van on miRNA-124 expression, decreasing its expression by 58.2% and 37.8% compared to their XAV939-free counterparts, respectively.

Similarly, miRNA-135 levels also showed significant difference between the groups (*F*_(6, 14)_ = 22.66, *P* < 0.0001, η2 = 0.906). miRNA-135 levels suffered a 73.5% decrease in Res-injected rats compared to the control group (Fig. 7B). The introduction of Miln and Van mitigated this effect and raised the miRNA-135 levels to a 3.26- and 3.29-fold compared to the FM group, respectively. On the other hand, both Miln + XAV939 and Van + XAV939 groups displayed 52.4% and 56.4% decrements in miRNA-135 levels compared to their XAV939-free counterparts, respectively.

### *Milnacipran and Vanillin Averted the Reserpine-Induced Decline in Rat Hippocampal 5-HT and 5-HT*_*1A*_* Receptor Protein Expression*

**Fig. 8:**
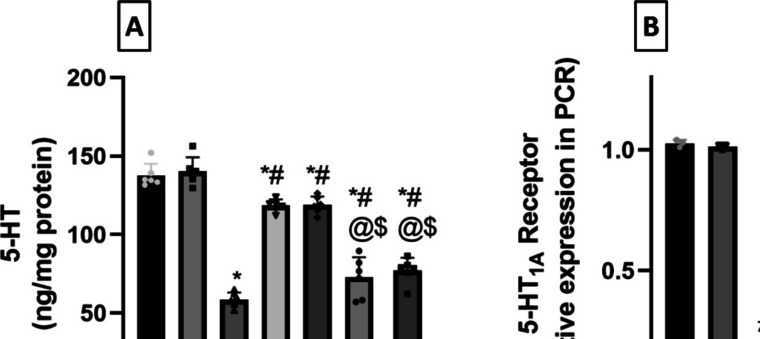
Effect of milnacipran and vanillin alone and in conjunction with XAV939 on reserpine-induced alterations in rat hippocampal **A** 5-HT level determined by ELISA and **B** 5-HT_1A_ receptor gene expression determined by qRT-PCR. Each bar with vertical line represents the mean ± SD for 5-HT (*n* = 6) and for 5-HT_1A_ (*n* = 3). *vs CTRL, #vs FM, ^@^ vs FM + Miln and ^$^ vs FM + Van using one-way ANOVA followed by Tukey’s multiple comparisons test; *P* < 0.05. CTRL, control; VAN, vanillin; FM, fibromyalgia; Miln, milnacipran; XAV, XAV939; 5-HT, 5-hydroxy tryptophan

The level of 5-HT varied significantly between the groups (*F*_(6, 35)_ = 105.8, *P* < 0.0001, η2 = 0.947). Res-challenged rats suffered from a reduction in the hippocampal levels of 5-HT (Fig. 8A) showing 57.5% significantly lower values compared to the control group. The administration of Miln and Van counteracted this effect raising 5-HT level to 2- and 2.03-fold, respectively, in comparison with the FM group. Wnt/β-catenin inhibition revoked the abovementioned effects of Miln and Van. This was manifested in Miln + XAV939 rats as a significant 38.6% decrement in 5-HT level compared to their XAV939-free counterparts. Similarly, Van + XAV939 rats displayed significant 35% reduction in 5-HT level compared to the XAV939-free Van group.

Furthermore, 5-HT_1A_ receptor protein expression was also significantly different between the tested groups (*F*_(6, 14)_ = 116.6, *P* < 0.0001, η2 = 0.98). The FM group exhibited a significant 79.5% decrease in 5-HT_1A_ expression compared to the control group (Fig. 8B). Miln and Van abrogated this decrease and increased the 5-HT_1A_ receptor protein expression to 3.66- and 3.84-fold, respectively, compared to the FM group. Co-administration of XAV939 with Miln and Van revoked their enhancing effect and resulted in 50.2% and 60.7% decrements in 5-HT_1A_ expression compared to their XAV939-free counterparts, respectively.

### Milnacipran and Vanillin Treatment Mitigated the Reserpine-Induced Neuronal Degeneration and Histological Aberrations in the Rat Hippocampal CA3 Region

**Fig. 9:**
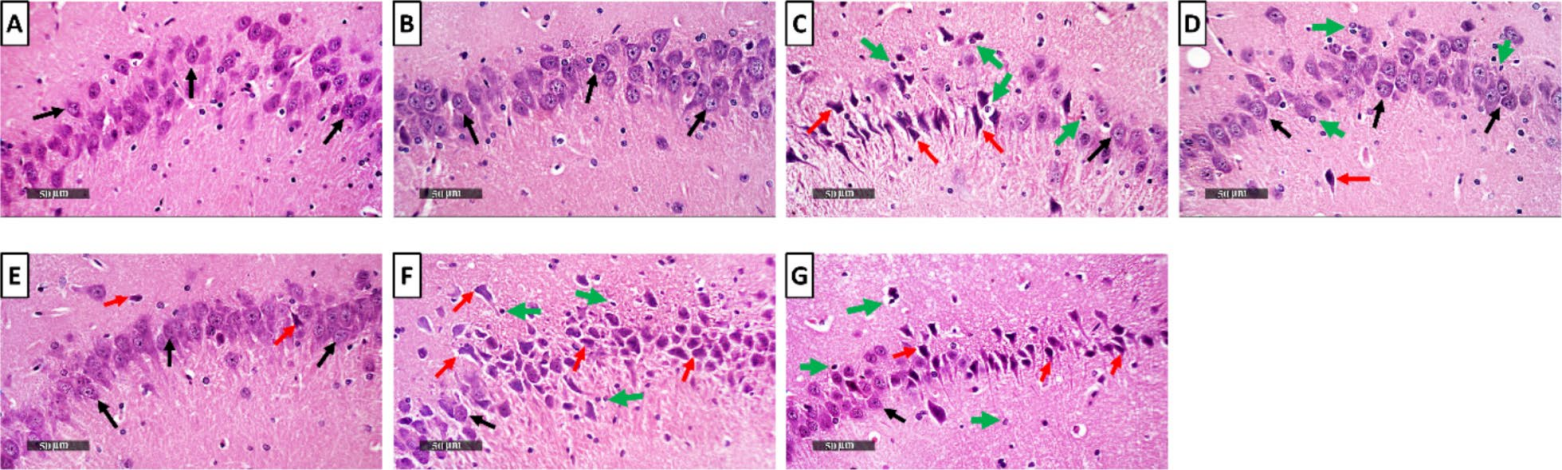
Effect of milnacipran and vanillin alone and in combination with XAV939 on the reserpine-induced histopathologic changes in the CA3 region of FM rats. **A–G** Histopathologic assessment using H&E stain at × 500 magnification: **A** representative photomicrograph of CA3 from the control group depicting normal organized morphological features of hippocampal layers with apparent intact pyramidal neurons demonstrating distinct nuclear and subcellular details (black arrow) with intact intercellular matrix. **B** Representative photomicrograph of CA3 from CTRL + Van group showing almost the same records as the control group samples without detectable abnormal histological changes. **C** Representative photomicrograph of CA3 from the Res group showing marked neuronal degenerative changes and loss with abundant figures of shrunken, angular and pyknotic neurons (red arrow) alternated with few intact cells (black arrow), accompanied with obvious perineuronal edema and higher records of reactive astrocytes as well as microglial cell infiltrates (green arrow). **D** Representative photomicrograph of CA3 from Res + Miln group showing evidence of significant neuroprotective efficacy (black arrow) with minimal records of neuronal damage (red arrow) with apparent intact intercellular brain matrix. However, persistent reactive glial cell infiltrates were observed (green arrow). **E** Representative photomicrograph of CA3 from Res + Van group showing almost the same records as Res + Miln group but with minimal records of glial cell infiltrates. **F** Representative photomicrograph of CA3 from Res + Miln + XAV939 group showing severe disorganization of hippocampal histological features with severe neuronal damage and loss (red arrow) with few intact pyramidal neurons (black arrow). Moreover, moderate to severe perineuronal edema and higher records of reactive astrocytes as well as microglial cell infiltrates were observed (green arrow). **G** Representative photomicrograph of CA3 from Res + Van + XAV939 group displaying similar records to the Res + Miln + XAV939 group

The control groups (Fig. 9A, B) exhibited typical and well-organized morphological characteristics of hippocampal layers with pyramidal neurons appearing intact. Distinct nuclear and subcellular details were displayed along with an intact intercellular matrix. The FM group (Fig. 9C) showed marked neuronal degenerative changes and loss with abundant figures of shrunken, angular, and pyknotic neurons interspersed with a few intact cells. Additionally, there was evident perineuronal edema and increased presence of reactive astrocytes and microglial cell infiltrates.

Conversely, the Miln group (Fig. 9D) exhibited substantial neuroprotective proficiency compared to the FM group. This was shown through minimal evidence of neuronal damage and a clear presence of an intact intercellular brain matrix. Nonetheless, persistent infiltration of reactive glial cells was observed. The Van group (Fig. 9E) demonstrated almost the same records as Miln samples, but minimal records of glial cell infiltrates were shown. XAV939 used concomitantly with Miln or Van (Fig. 9F, G) produced similar results to one another and to the Res group. This was exhibited by severe disorganization of hippocampal histological features with severe neuronal damage and loss with few intact pyramidal neurons. Moreover, moderate to severe perineuronal edema and higher records of reactive astrocytes as well as microglial cell infiltrates were detected. The histopathological scoring of neuronal degenerative changes, and glial cell infiltration, as well as brain matrix vacuolization and edema in CA3 are presented in Table 2.

**Table 2:** Histopathological scoring of hippocampal lesions; neuronal degenerative changes, and glial cell infiltration, as well as brain matrix vacuolization and edema in CA3 and DG

CA3							
Groups Lesions	**CTRL**	**CTRL VAN**	**FM**	**FM + MILN**	**FM + VAN**	**FM + MILN + XAV939**	**FM + VAN + XAV939**
Neuronal degenerative changes	0	0	3	1	1	3	3
Glial cell infiltrates	0	0	2	2	0	2	2
Brain matrix vacuolization/edema	0	0	3	0	0	3	3
DG							
Groups Lesions	**CTRL**	**CTRL VAN**	**FM**	**FM + MILN**	**FM + VAN**	**FM + MILN + XAV939**	**FM + VAN + XAV939**
Neuronal degenerative changes	0	0	3	1	1	3	3
Glial cell infiltrates	0	0	1	0	0	1	1
Brain matrix vacuolization/edema	0	0	1	1	1	1	1

The 4-point scoring system is defined as follows: 0 = negative record, 1 = mild records in less than 15% of examined tissue sections, 2 = moderate records in 16–35% of examined tissue sections, 3 = severe records in more than 35% of examined tissue section. *CTRL* control, *VAN* vanillin, *FM* fibromyalgia, *Miln* milnacipran, *XAV* XAV939

**Fig. 10:**
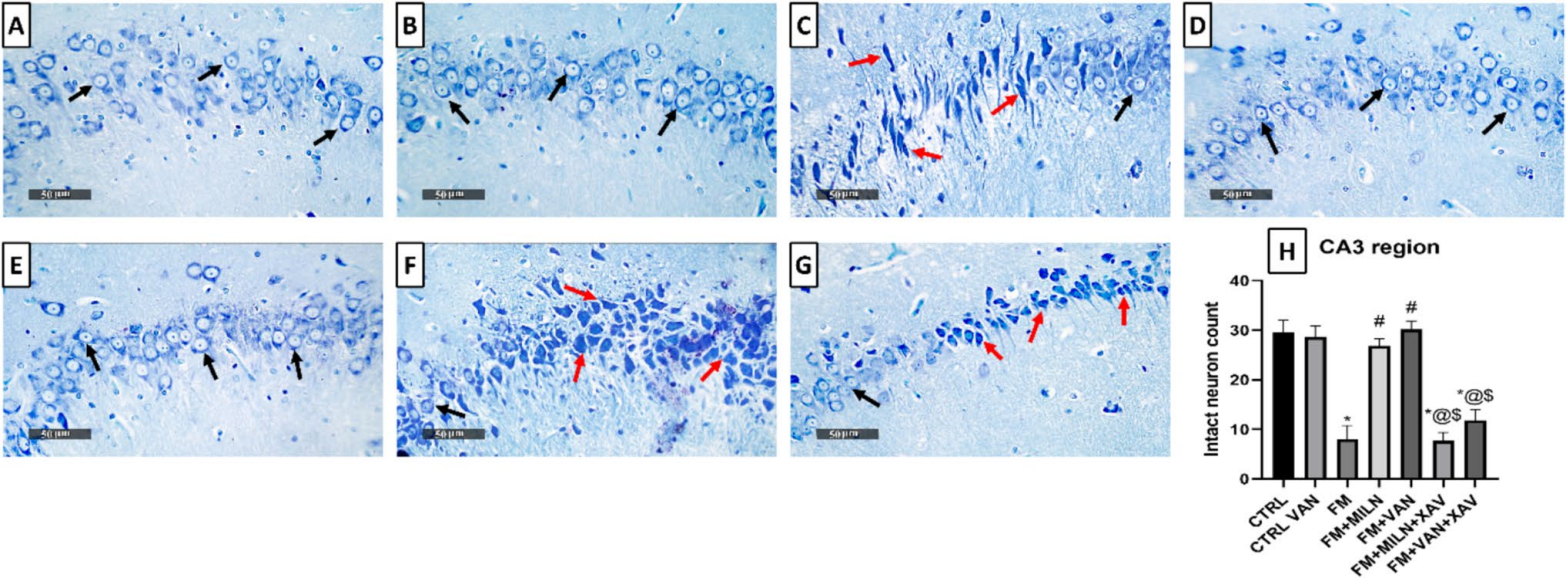
Effect of milnacipran and vanillin alone and in combination with XAV939 on the reserpine-induced neuronal damage in the CA3 region of FM rats. **A–H** Neuron visualization by Nissl staining (toluidine blue) at × 500 magnification. **A**, **B** Representative photomicrographs of CA3 from the CTRL and CTRL + Van groups, respectively, showing intact neurons (black arrow). **C** Representative photomicrograph of CA3 from the reserpine group showing a large proportion of degenerated neurons (red arrow). **D**, **E** Representative photomicrographs of CA3 from the Res + Miln group and reserpine + vanillin group, respectively, showing preserved neuronal integrity with a marked reduction of the degenerative neuronal records and higher records of intact neurons (black arrow). **F**, **G** Representative photomicrographs of CA3 from the Res + Miln + XAV939 and Res + Van + XAV939 groups, respectively, showing a large proportion of degenerated neurons (red arrow) with marked reduction in intact neurons (black arrow). **H** Assessment of the intact neuron count in CA3 of Nissl-stained sections. Data are expressed as the mean ± SD of number of intact neurons/field for six non-overlapping fields/section; *n* = 3; *vs CTRL, #vs FM, ^@^ vs FM + Miln and.^$^ vs FM + Van (one-way ANOVA followed by Tukey’s multiple comparisons test; *P* < 0.05). CTRL, control; VAN, vanillin; FM, fibromyalgia; MILN, milnacipran; XAV, XAV939

Examining neuronal integrity by Nissl staining (Fig. 10A, H), FM rats displayed a marked 73% decrement in the number of intact neurons in the CA3 region, compared to the control group. This decrease was abrogated by Miln and Van administration which increased the neuronal count to 3.35- and 3.77-fold compared to the FM group, respectively. The use of XAV939 either with Miln or Van resulted in a 71.4% and 68.8% decrease in neuronal count contrasted to their XAV939-free counterparts, respectively.

### Milnacipran and Vanillin Treatment Attenuated the Reserpine-Induced Neuronal Degeneration and Histological Aberrations in the Rat Hippocampal Dentate Gyrus Region

**Fig. 11:**
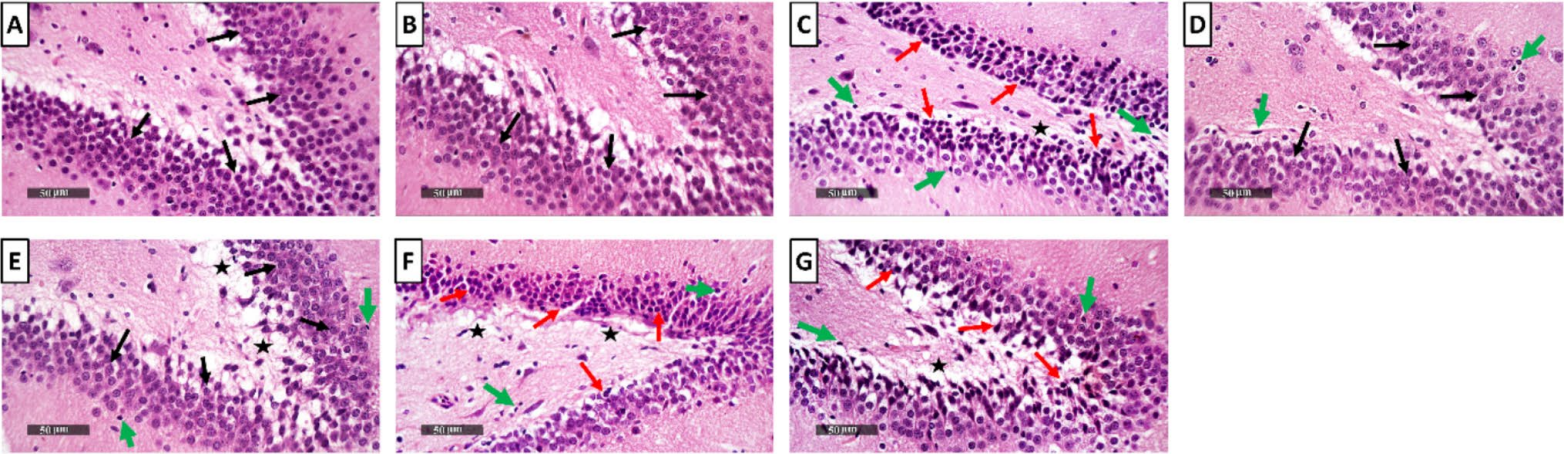
Effect of milnacipran and vanillin alone or in combination with XAV939 on the reserpine-induced histopathologic changes in the dentate gyrus of FM rats. **A–G** Histopathologic assessment using H&E stain at × 500 magnification: **A**,** B** Representative photomicrographs of dentate gyrus from the control and control + vanillin groups, respectively, depicting normal histological structures of hippocampal layers including granule cells at different zones of hippocampal apexes and blades with almost intact subcellular details (black arrow) as well as hilar region without abnormal alterations and minimal glial cell infiltrates. **C** Representative photomicrograph of the dentate gyrus from the reserpine group showing abundant figures of degenerated granule neurons with pyknotic nuclei allover different zones of the dentate gyrus (red arrow) and mild intercellular edema and vacuolization of brain matrix (star), accompanied with mild higher reactive glial cell infiltrates than the control and control + vanillin groups (green arrow). **D** Representative photomicrograph of dentate gyrus from reserpine + milnacipran group showing significant higher neuroprotective efficacy than the reserpine only group with almost intact well organized granule cells (black arrow), single sporadic neuronal damage records with mild vacuolization of brain matrix and minimal reactive glial cell infiltrates (green arrow). **E** Representative photomicrograph of dentate gyrus from reserpine + vanillin group showing almost the same records as reserpine + milnacipran samples but with persistent vacuolization of brain matrix shown in inner aspect granule cells (star). **F**,**)** Representative photomicrographs of dentate gyrus from reserpine + milnacipran + XAV939 and reserpine + vanillin + XAV939 groups, respectively, showing similar records to the disease group

The control samples demonstrated typical histological architecture of hippocampal layers (Fig. 11A, B). This includes granule cells at different zones of hippocampal apexes and blades with almost intact subcellular details. Additionally, the hilar region showed no aberrant alterations and minimal glial cell infiltrate. On the other hand, the FM group exhibited numerous figures of degenerated granule neurons with pyknotic nuclei all over the different zones of dentate gyrus (Fig. 11C). Additionally, mild intercellular edema and vacuolization of brain matrix were displayed accompanied with mild higher reactive glial cell infiltrates compared to the control groups. Miln group (Fig. 11D) displayed significant neuroprotective efficacy compared to the FM group with almost intact well-organized granule cells and single sporadic neuronal damage records. The Miln group also exhibited mild vacuolization of brain matrix with minimal reactive glial cell infiltrates. The Van group (Fig. 11E) showed similar results to the Miln group in addition to persistent vacuolization of brain matrix in inner aspect granule cells. The Miln + XAV939 and Van + XAV939 groups showed similar records to those of the FM group (Fig. 11F, G). The histopathological scoring of neuronal degenerative changes, and glial cell infiltration, as well brain matrix vacuolization and edema in DG are presented in Table 2.

**Fig. 12:**
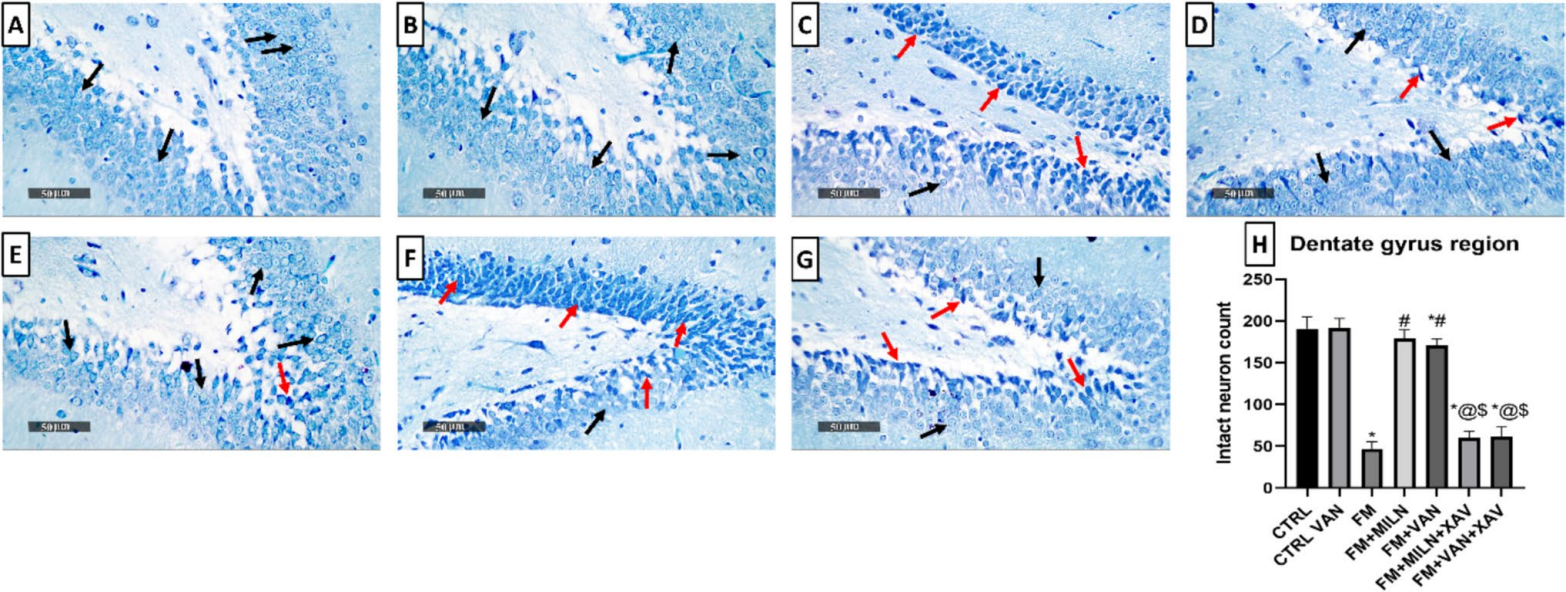
Effect of milnacipran and vanillin alone or in combination with XAV939 on the reserpine-induced neuronal damage in the dentate gyrus of FM rats. **A–H** Neuron visualization by Nissl staining (toluidine blue) at × 500 magnification. **A**, **B** Representative photomicrographs of dentate gyrus from the control and control + Van groups, respectively, showing intact neurons (black arrow). **C** Representative photomicrograph of dentate gyrus from the Res group showing a large proportion of degenerated neurons (red arrow). **D**, **E** Representative photomicrographs of dentate gyrus from the Res + Miln group and Res + Van group, respectively, showing preserved neuronal integrity with a marked reduction of the degenerative neuronal records (red arrow) and higher records of intact neurons (black arrow). **F**, **G** Representative photomicrographs of dentate gyrus from the Res + Miln + XAV939 and Res + Van + XAV939 groups, respectively, showing a large proportion of degenerated neurons (red arrow) with marked reduction in intact neurons (black arrow). **H** Assessment of the intact neuron count in dentate gyrus of Nissl-stained sections. Data are expressed as the mean ± SD of number of intact neurons/field for six non-overlapping fields/section; *n* = 3; *vs CTRL, #vs FM, ^@^ vs FM + Miln and.^$^ vs FM + Van using one-way ANOVA followed by Tukey’s multiple comparisons test; *P* < 0.05. CTRL, control; VAN, vanillin; FM, fibromyalgia; MILN, milnacipran; XAV, XAV939.

Evaluation of neuronal integrity via Nissl staining revealed that the FM group displayed a significant 75.4% reduction in the intact neuron count in the dentate gyrus, compared to the control group. Miln and Van administration counteracted this decrease by increasing the neuronal count to 3.81- and 3.63-fold compared to the FM group, respectively. The use of XAV939 either with Miln or Van resulted in a decrease in neuronal count by 66.3% and 63.6% respectively, compared to their XAV939-free counterparts (Fig. 12A, H).

## Discussion

To the best of our knowledge, the biochemical and behavioral results demonstrated in the present investigation exhibit the first evidence for the neuroprotective, analgesic, and antidepressant role demonstrated by Van in a female rat model of FM. The results indicate that the antidepressant effect of Van and Miln may be mediated by the canonical Wnt signaling pathway. This is supported by multiple lines of evidence: (i) reduction in the depressive like behavior in forced swim test, (ii) improvement of FM hallmarks like motor coordination and pain in behavioral tests, (iii) reversal of the Reserpine (Res)-induced decrease in various players of the Wnt/β-catenin signaling pathway, (iv) marked increase in certain key stress resilience miRNAs, and (v) improved histological architecture and intact neurons of the hippocampus. The mechanistic impacts of these drugs were evaluated using a well-established inhibitor of the canonical Wnt signaling in several experimental models, XAV939.

FM presents with functional and structural alterations in the CNS, particularly affecting the brain regions involved in pain processing [68, 69]. Reserpine-induced FM (RIF) is an exemplary biochemical model. It replicates the pathological and clinical manifestations of FM. This includes depletion of biogenic amines, oxidative stress, depressive symptoms, and musculoskeletal pain without evident tissue damage, mirroring the clinical observations in FM patients [15]. The Res model typically demonstrates widespread pain in the form of hyperalgesia and allodynia, which is pain due to a normally non-pain-provoking stimulus [70]. The privilege beyond utilization of Res-induced FM is the production of allodynia, hyperalgesia, and depression analogous to the clinically manifested FM symptoms [15, 71]. Considering the psychiatric domain, studies have shown that FM patients with comorbid depression were more sensitive to pain than non-depressed ones [6, 8, 9]. This further proves pain and depression are intimately related and suggests that either depression may decrease the pain threshold or that it is a normal product of the pain. Thus, the antinociceptive character exhibited by Van and Miln may be, in part, owed to their antidepressant effects which maybe owed to the activation of Wnt/β-catenin signaling. In the present investigation, RIF-inflicted depression was tested through the forced swim test. The Res group showed significant increase in the immobility time in concordance to former research [72–74]. On the other hand, both Miln and Van shortened the immobility time significantly indicating antidepressant effect. The antidepressant effect of Miln in FST had previously been recorded in a murine model of depression [75] while that of Van has been reported to be comparable to fluoxetine [44]. Abo-youssef pointed out the impact of Van on stress-evoked behavioral and biochemical alterations via elevating brain 5-HT level, shortening FST immobility time and elevating the consumption of sucrose in the sucrose preference test [52]. The antidepressant effects of both Miln and Van were diminished upon the co-administration of XAV939 which further implicated the Wnt/β-catenin cascade in their antidepressant mechanism.

Res-induced pain assessment has been previously verified using different behavioral tests [51]. The present study tended to verify the induction of FM model by assessing the pain behavioral tests. In the present study, the Res FM rat model exhibited hyperalgesic and allodynic behaviors. This was evidenced by the significant decrease in withdrawal threshold in the Randall-Selitto, tail immersion, hot plate, and cold allodynia tests, as compared to the control group. Such decline in withdrawal threshold was parallel to a significant drop in tension tolerance in the Von Frey test as indication of mechanical allodynia. Nevertheless, treatment with either Van or Miln significantly ameliorated these hyperalgesic and allodynic effects. The analgesic effect of Miln in FM models had been previously witnessed [35, 76, 77]. On the other hand, this is the first report of Van ameliorating the hyperalgesia and allodynia in a FM rat model, despite showing anti-nociceptive effects in mice subjected to the hot plate test [78], in carrageenan-induced edema model [45] and the acetic acid writhing test [79]. Moreover, Van depressed allodynia inflicted by chronic constriction of sciatic nerve [80]. The present findings indicate that Van administration did not produce any observable behavioral and biochemical changes in control rats. This lack of effect aligns with the understanding of Van as a food supplement with recognized biological activity that primarily manifests in case of induced stress or pathological conditions, rather than in baseline, and healthy models. Bhagwat et al. pointed to this understanding, as Van exhibits anxiolytic effects only in the presence of environmental stressors, suggesting its activity may be condition-dependent. The anxiolytic effect of Van in rats was only when exposed to unfamiliar, brightly lit environments that naturally provoke stress responses [81]. However, in the present study, the controlled environmental conditions, with strict regulation of these variables, may explain the lack of effect of Van in the control animals. Further evidence supported this notion where Van does not significantly impact normal physiological and biochemical parameters in 3-nitroprpionic acid model. In a study administering Van at 150 mg/kg orally for 19 days, no significant changes were observed in body weight, locomotor activity, motor coordination, spatial navigation, or even mitochondrial markers, such as brain striatum TBARS, nitrite/nitrate levels, glutathione, superoxide dismutase, catalase, or acetylcholinesterase activity. However, Van presented protective role for these parameters after 3-nitropropionic acid aberrations [82]. Van similarly showed crucial mitochondrial protection and neuroprotective actions [83]. Additionally, although of provoking unconscious state in rats, research on high-dose of Van has demonstrated that it is not toxic when administered orally or intraperitoneally at 150–300 mg/kg, and it may even provide blood and brain protective properties. This is further confirmed by genomic analysis and maintained normal expression levels of genes related to DNA damage, and inflammation, indicating potential neuroprotective effects [84]. Given these findings, the present study concludes that Van’s effects are largely evident in stress-induced or disease conditions and not in normal models, which aligns with the present observations of no behavioral or biochemical changes in healthy control animals. More future studies should demonstrate the rationale behind Van’s effect in pathological conditions rather than non-pathological one, providing deeper illustrations about its specific mechanism of action.

FM is originally a musculoskeletal disorder characterized by motor anomalies. Interestingly, Miln enhanced motor coordination in RIF rats as shown by spending more time on the rotating rod which may be, in part, due to the alleviation of muscle and joint pain. Interestingly, Miln’s boosting effect on motor coordination was antagonized by the co-administration of XAV939, which may implicate the Wnt/β-catenin signaling pathway. On the other hand, Van treatment led to a slight increment in the duration spent by the rats holding the rotarod in comparison with the Res group, but this increase did not mount to any significance. We could, therefore, conclude that although Van has a dopaminergic action that is effective in the amelioration of motor disturbance in Parkinson’s disease [85, 86], it has no beneficial effect on FM-induced motor incoordination.

Wnt/β-catenin and the serotonergic system are the cornerstone in the pathophysiology of stress-associated depression. Brain specimens from individuals with major depressive disorder revealed abnormalities in Wnt signaling activity [18]. Moreover, β-catenin gene excision in the nucleus accumbens increased the vulnerability of mice to chronic stress induced by social defeat. Dias and co-workers reported that behavioral resilience in mice is promoted by β-catenin through control of its downstream target, dicer enzyme, which produces stress-resistant miRNAs [19]. The level of β-catenin is mastered by the status of Wnt and GSK-3β signaling. Once Wnt binds to the Frizzled transmembrane receptor and its co-receptors, the phosphorylated β-catenin is no longer ubiquitinated nor degraded. This results in cytoplasmic saturation with newly synthesized β-catenin followed by translocation into the nucleus where it serves as a co-activator and binds with TCF/LEF transcription factors. This facilitates the transcription of target genes associated with the Wnt pathway [17, 18] such as neurogenin1 and NeuroD1 which regulate neuronal differentiation [87, 88]. On the other hand, in the Wnt off state, the active unphosphorylated form of GSK-3β phosphorylates β-catenin as a preapprehension to its degradation [18, 89]. Thus, chronic stress is accompanied with elevated active GSK-3β levels which in turn decreases β-catenin [17, 89]. In rodents, studies report that decreased β-catenin levels are associated with increased anxiety and fear-like behavior, thus correlating increased levels of β-catenin with higher suppression of stress behavior [19]. This suggests the possible protective effect of β-catenin in depression. The same conclusion was noted when sulindac enhanced stress-like behavior after downregulating β-catenin expression. On the contrary, the lithium-induced upregulation of the Wnt pathway suppressed the stress behavior [22]. The present work seeks to link the FM co-morbid stress and depression to the Wnt/β-catenin cascade. The injection of Res incited a significant reduction in Wnt and phosphorylated GSK-3β levels which subsequently decreased β-catenin, TCF, and dicer levels in rodents’ hippocampi compared to the control rats. This study hypothesizes that the depression associated with FM may be a result of the diminished expression of the end products of the canonical Wnt signaling. The downregulation in the pathway effectors brought about by Res was mitigated significantly upon the introduction of either Miln or Van. Miln and Van treatments both resulted in marked increments in hippocampal Wnt, the p-GSK-3β/t-GSK-3β ratio, β-catenin, TCF, and dicer levels, compared to the Res-challenged rats. The boosting effect exerted by Miln and Van on the Wnt/β-catenin axis was antagonized by XAV939 addition.

β-Catenin is influenced by neurotransmitters such as 5-HT and dopamine. This was observed with serotonergic antidepressants, such as fluoxetine and citalopram, where both altered β-catenin levels [18]. Phosphorylated-GSK-3β levels are mastered by 5-HT_1A_ and 5-HT_2_ receptors as shown when a 5-HT_1A_ agonist in the hippocampus increased the level of p-GSK-3β, while that of 5-HT_2_ decreased it [27, 28]. Miln is a serotonin and nor-epinephrine reuptake inhibitor (SNRI); thus, its herein observed effect on 5-HT and, consequently, on p-GSK-3β and β-catenin was expected. The antidepressant effect of Van, as evidenced by the FST results in this study, had previously been attributed in part to the agonistic action on α_2_ adrenergic receptors in a previous study of stress-induced depression in mice [81]. Another finding by Xu and co-workers reported that Van raises 5-HT in the brain, sequentially alleviating chronic depressive symptoms which is in concordance with the present findings [48]. This may interpret the effects of Miln and Van on Wnt pathway as being attributed to their serotonergic activation in the hippocampus. To further validate this theory, the study assessed the levels of 5-HT and 5-HT_1A_ receptor expression and revealed a significant increase in the Miln and Van treatment groups compared to the Res-challenged group. The blockade of the Wnt pathway by XAV939 counteracted these elevations.

While the precise mechanisms linking the decrease in β-catenin with the emergence of depressive symptoms remain largely understudied, it has been proposed that a significant role may involve the stress resilience miRNAs produced by dicer enzyme such as miRNA-124 and miRNA-135. In a study by Issler and co-workers, it was found that miRNA-135 levels in the blood of depressed patients increased after antidepressant treatment. They also proved that miRNA-135 overexpression conferred resilience to chronic social defeat stress via targeting mRNAs that encode the 5-HT transporter and 5-HT_1A_ receptor [90]. This miRNA-135-induced modulation causes an increase in 5-HT level in the synaptic cleft, which is associated with a decrease in depressive symptoms. A recent study discovered miRNA-135 was downregulated in peripheral blood in patients with major depressive disorder [91, 92]. In the present investigation, the expression of hippocampal miRNA-135 in the FM like model was notably depressed when compared with the control group. This Res-induced decrease in miRNA-135 expression was mitigated significantly in both the Miln and Van groups which may be ascribed to their activating role in the Wnt signaling pathway. This was further proven upon the administration of XAV939 which abrogated the protective effect exerted by Miln or Van for miRNA-135. It is worthy to note that the observed alterations in miRNA-135 levels were coupled with concordant alterations in 5-HT levels and 5-HT_1A_ receptor expression in the hippocampus, which is in accordance with the formerly reported miRNA-135-mediated modulation of 5-HT [90].

Additionally, numerous investigations indicate the direct involvement of miRNA-124 in resilience/susceptibility to stress and depressive behavior. In a murine model of chronic ultra-mild stress, an increase in depression-like behavior and a reduction in hippocampal expression of miRNA-124 were reported. Such effect was mitigated by long-term treatment with imipramine, a tricyclic antidepressant [93]. Higuchi and co-workers also reported that increasing miRNA-124 hippocampal levels via viral-mediated overexpression imparted behavioral resilience to stressed mice, while halting miRNA-124 resulted in increased vulnerability to stress-related behaviors. This indicates that reducing hippocampal miRNA-124 seems to heighten vulnerability to depression-like behaviors following exposure to mild chronic stress. Higuchi and colleagues then proposed that miRNA-124’s role in stress resilience was mediated by its post-transcriptional regulation of mRNA or protein expression levels of histone deacetylases HDAC4/5 and GSK-3β, all of which are highly conserved miRNA-124 targets. However, these results are in contrast with the increased expression levels of miRNA-124 in the hippocampus and basolateral amygdala of stressed animals reported in previously described studies [94–96]. These discrepancies might be due to differences in the animal species, the stress paradigm, or the genetic background. This also indicates that the cell type may have an influence on the role of miRNA-124 in stress/depression. However, it is clear from the aforementioned studies that miRNA-124 is robustly linked with depression. The herein reported findings revealed a marked decrease in the hippocampal level of miRNA-124 in the Res group that corresponded to the depressive behavior shown by that group in the FST. On the other hand, Miln and Van both increased hippocampal miRNA-124 level significantly which agrees with Higuchi and co-workers’ findings that antidepressant treatment increases the level of this miRNA [93]. The implication of the Wnt/β-catenin pathway in the replenishment of miRNA-124 was further validated by its diminished levels in the groups which received XAV939.

Notably, the unusual variation of the data for miRNA-124 and miRNA-135 can be attributed to several factors related to the biological and pharmacological effects of Res, oxidative stress, and XAV939 on miRNA regulation. The control groups exhibited low variability, as expected since these groups were not exposed to Res, a known stress-inducing agent, where their miRNA levels remained stable and showed minimal variability. In contrast, the Res-treated groups exhibited significant variability in miRNA expression. Res, a monoamine-reducing agent, provokes substantial stress and oxidative stress by inhibiting the vesicular monoamine transporter. This inhibition enhances monoamine turnover resulting in reactive oxygen species (ROS) production that disrupts DNA, and normal cellular functions, particularly miRNA expression patterns [97–99]. ROS have been shown to modulate transcription factors involved in miRNA biogenesis, resulting in stress-induced dysregulation of miRNA at various levels [100]. Additionally, stress can lead to the formation of stress granules that sequester specific miRNAs and their mRNA targets, altering their availability and functional activity within the cell [101]. These mechanisms collectively contribute to the observed variability in miRNA expression among the Res-treated groups. However, the variability was particularly pronounced in the XAV939-treated groups, a phenomenon consistent with findings by El-Kadi et al., 2024 [58]. Their study reported higher standard deviation in miRNA expression levels in XAV939-treated rats compared to control groups. XAV939, a tankyrase inhibitor, disrupts the Wnt/β-catenin signaling pathway, which plays a critical role in stress response regulation and cellular signaling. The disruption of this pathway likely interacts with Res-induced oxidative stress to further exacerbate miRNA variability. These combined effects underscore the complex interplay between oxidative stress, pharmacological interventions, and miRNA regulation in these experimental groups.

FM is accompanied by reduction of hippocampal volumes in humans as previously reported [102, 103]. RIF also disrupts the normal histological architecture in rodents’ spinal cords [72, 104], substantia nigra [71], cerebral cortex [105], and DG [106]. The present study is in alignment with these previous reports, displaying marked neuronal degenerative damage confirmed by toluidine blue stain, perineuronal edema, vacuolization, and architectural loss in the CA3 and DG hippocampal regions in Res-challenged rats, in comparison with the control group. These effects were ameliorated by Miln and Van which reversed the Res-induced architectural damage and elevated the intact neuronal count in CA3 and DG, compared to the FM group, showing evidence of neuroprotective efficacy, and further supporting our biochemical and molecular findings.

Further studies may be needed to confirm the role of the Wnt/β-catenin pathway in the alleviation of depression. While the present study focused on the hippocampus, it may be worthwhile to explore the role of this pathway in other areas of the brain and detect other stress resilience miRNAs. Moreover, further investigations are recommended to elucidate the discrepancy between effects of Van and Miln on the motor domain. Also, the study acknowledges that there is concern regarding reserpine’s dual induction of FM and depression, and about its suitability for modeling comorbidities. However, the dosage and duration of Res administration required to induce depression and FM differ significantly. This distinction supports the interpretation that depression in this model arises as a comorbidity of FM rather than as a direct effect of reserpine itself. In the present study, subcutaneous (s.c.) administration of reserpine at a dose of 1 mg/kg daily for 3 days was utilized. This protocol was carefully chosen to induce FM followed by depression without prolonged or excessive reserpine exposure. This approach differs from other studies that used Res exclusively to induce a depressive phenotype through longer administration periods and intraperitoneal (i.p.) injections. For example, some studies used Res at 0.2 mg/kg daily for 14 days [107] or over a 20-day period with low (0.2 mg/kg) or high (0.8 mg/kg) doses [108]. Other protocols involved i.p. injections of 0.2 mg/kg/day for 14–21 days [109, 110] higher doses such as 1.5 mg/kg i.p. for 10 days have also been employed [111, 112]. These studies demonstrate that prolonged reserpine administration is commonly used to develop a depressive model.

**Fig. 13:**
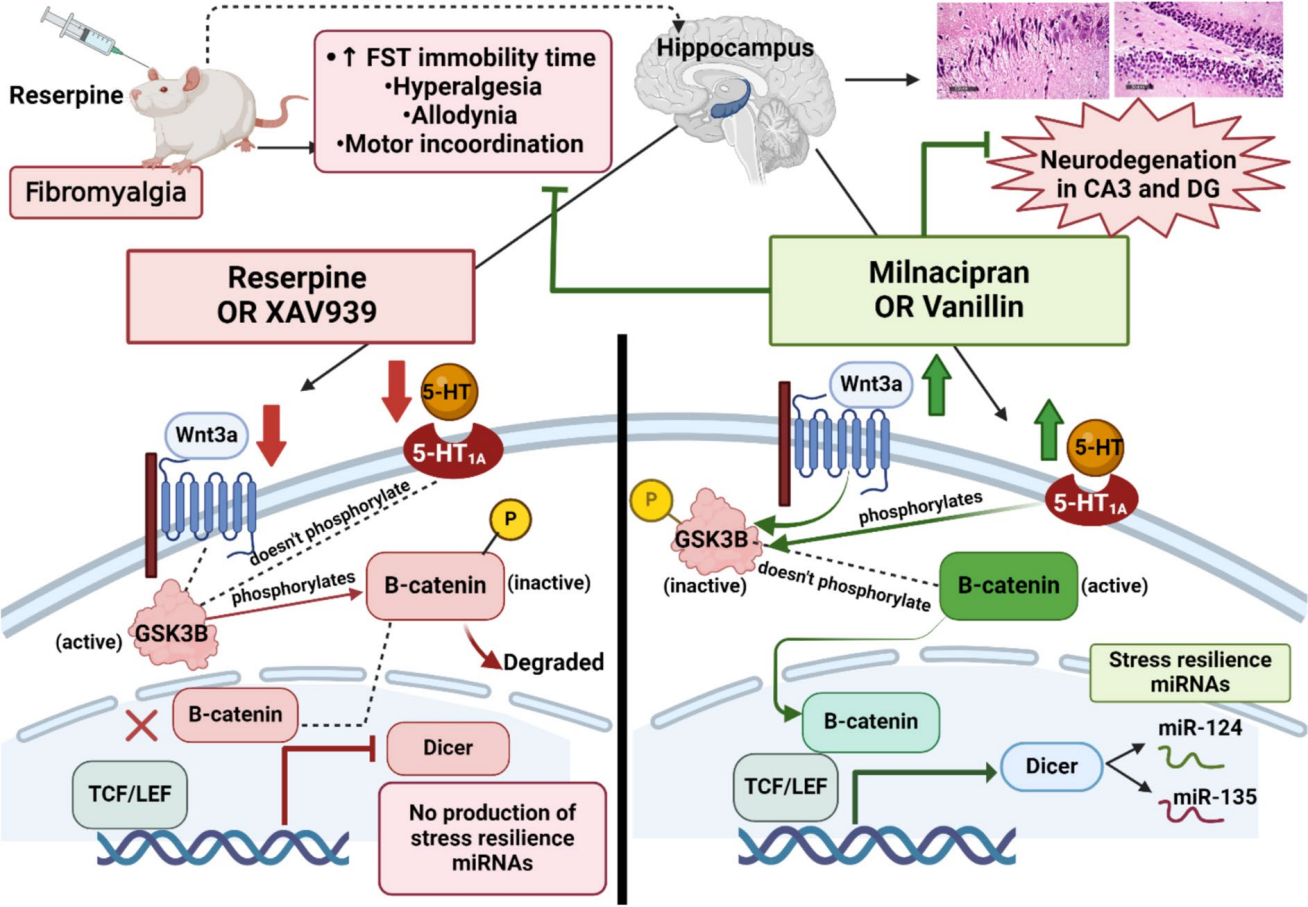
Schematic representation summarizing the mechanisms underlying the antidepressant effects of vanillin and milnacipran in reserpine-induced fibromyalgia in rats. 5-HT, 5-hydroxytryptophan; 5-HT_1A_, 5-hydroxytryptophan receptor_1A_; DG, dentate gyrus; FST, forced swim test; GSK-3β, glycogen synthase kinase-3beta; TCF, T cell factor; TCF/LEF, T cell factor/lymphoid enhancer-binding factor; Wnt, wingless-related integration site

In conclusion, as summarized in Fig. 13, the current study showed, for the first time, the ameliorative effect of Van on the depression associated with FM disease compared to Miln which is a standard FDA-approved marketed drug for FM. These favorable effects were proven to be mediated by the activation of Wnt/β-catenin signaling by both drugs, thereby resulting in a downstream upregulation of stress resilience miRNAs 124 and 135 which impart an antidepressant/stress-resilient effect on the rodents.

## Supplementary Information

Below is the link to the electronic supplementary material.

Supplementary file1 (DOCX 1599 KB)

## Data Availability

No datasets were generated or analysed during the current study.

## Abbreviations

5-HT: 5-Hydroxytryptophan
5-HT_1A_: 5-Hydroxytryptophan receptor_1A_
β-actin: Beta-actin
β-catenin: Beta-catenin
DG: Dentate gyrus
DMSO: Dimethyl sulfoxide
FM: Fibromyalgia
FST: Forced swim test
GSK-3β: Glycogen synthase kinase 3-beta
HDAC: Histone deacetylases
HPWL: Hind paw withdrawal latency
Miln: Milnacipran
PWL: Paw withdrawal latency
PBS: Phosphate-buffered saline
Res: Reserpine
RIF: Reserpine-induced fibromyalgia
RIPA: Radioimmunoprecipitation assay
RRFOL: Average rotarod fall-off latency
SNRI: Serotonin and nor-epinephrine reuptake inhibitor
TBST: Tris-buffered saline with Tween 20
TCF: T cell factor
TCF/LEF: T cell factor/lymphoid enhancer-binding factor
Van: Vanillin
Wnt: Wingless-related integration site
