# Supplementary material for: Milnacipran and Vanillin Alleviate Fibromyalgia-Associated Depression in Reserpine-Induced Rat Model: Role of Wnt/β-Catenin Signaling
Source: Mol Neurobiol. 2025 Feb 10;62(6):7682–705. doi: 10.1007/s12035-025-04723-w (PMC12078381; doi:10.1007/s12035-025-04723-w)
Supplement: Supplementary file 1 — Supplementary file1 (DOCX 1599 KB) [file 12035_2025_4723_MOESM1_ESM.docx]

**Beta-actin**

**
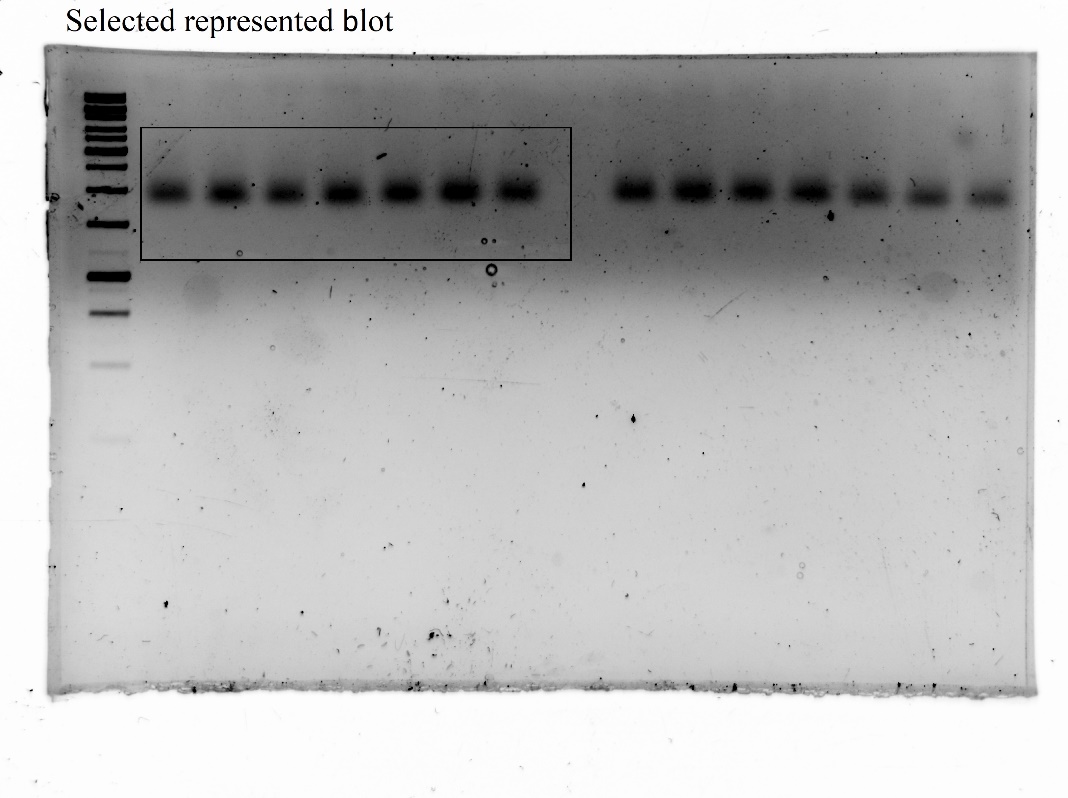
**

Samples 1-2

**
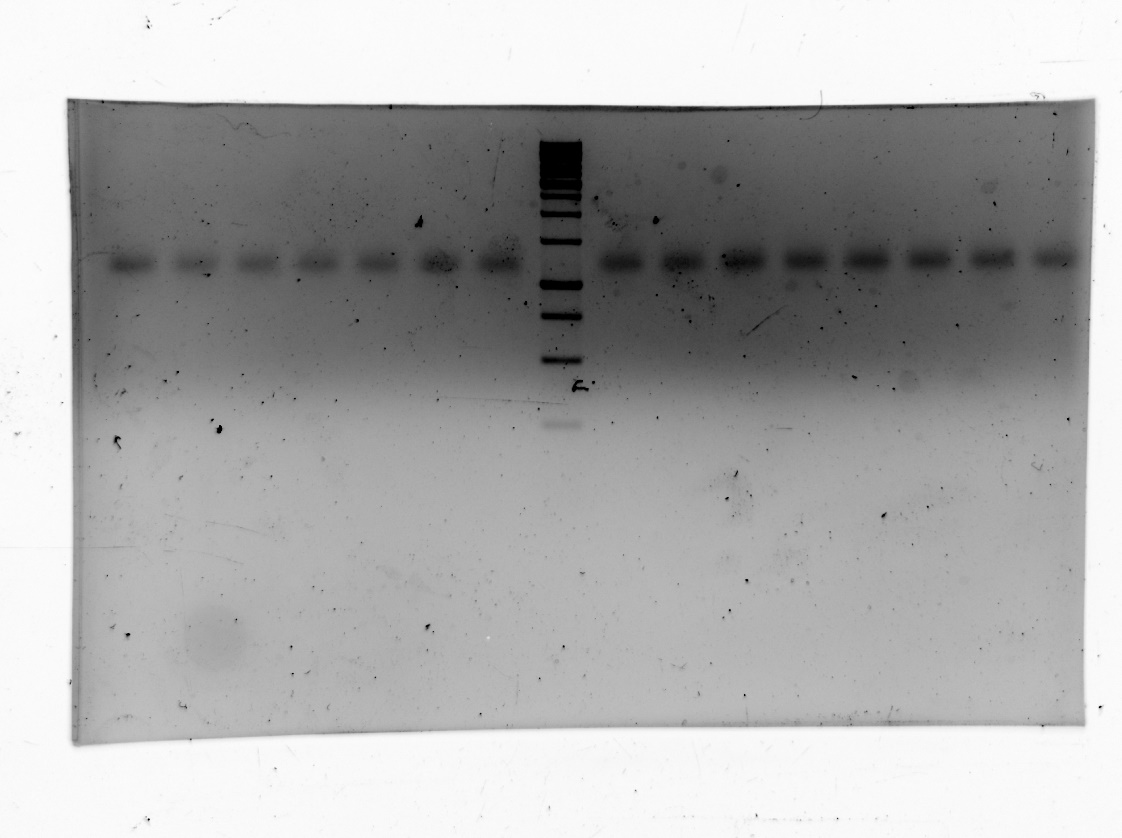
**

Samples 3-4

**Wnt3a**

**
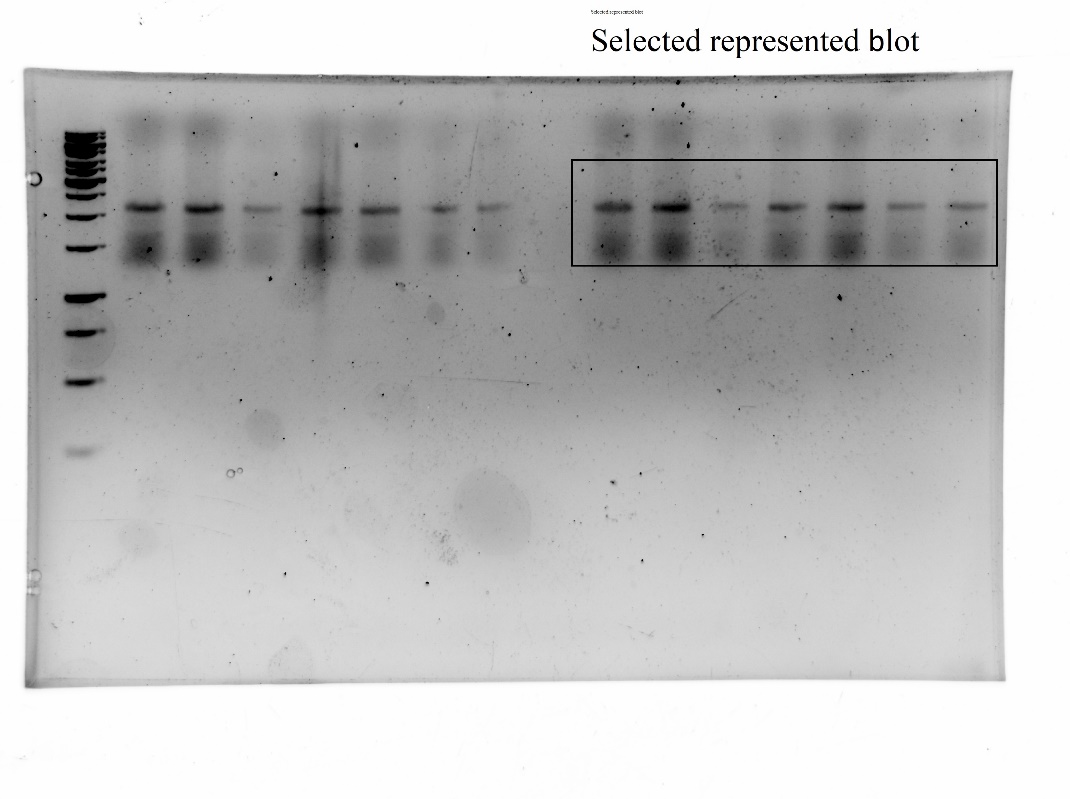
**

Samples 1-2

**
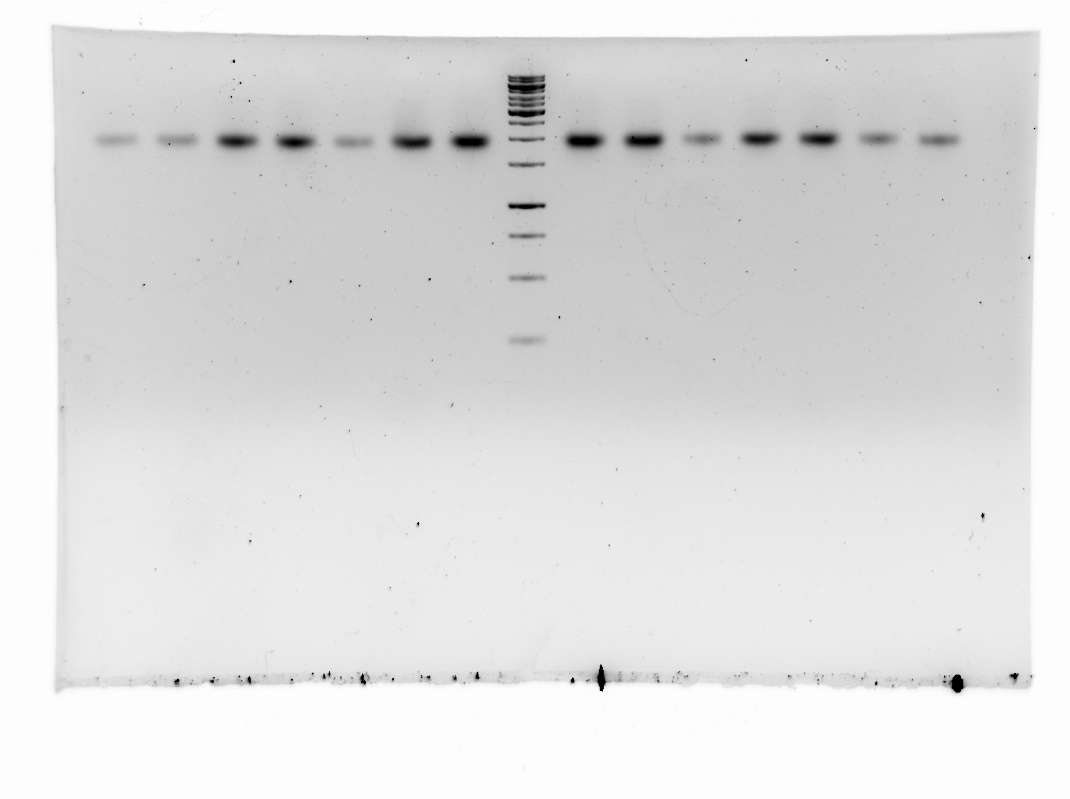
**

Samples 3-4

**Phosphorylated GSK**

**
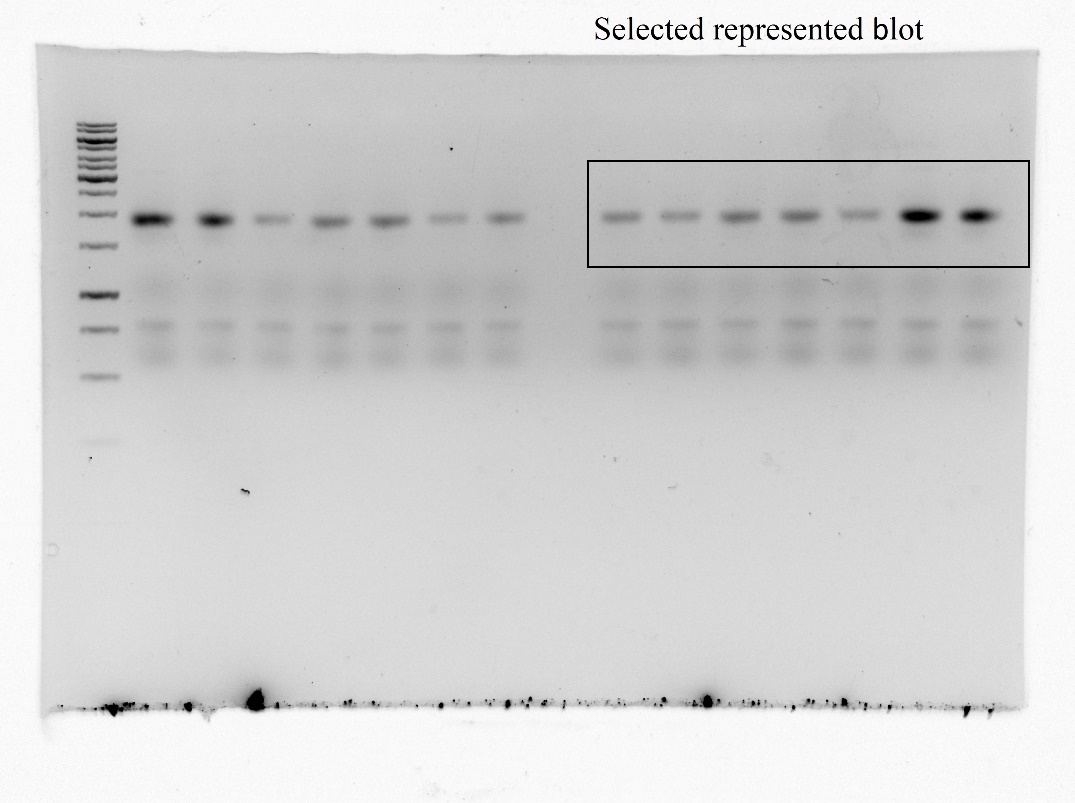
**

Samples 1-2

**
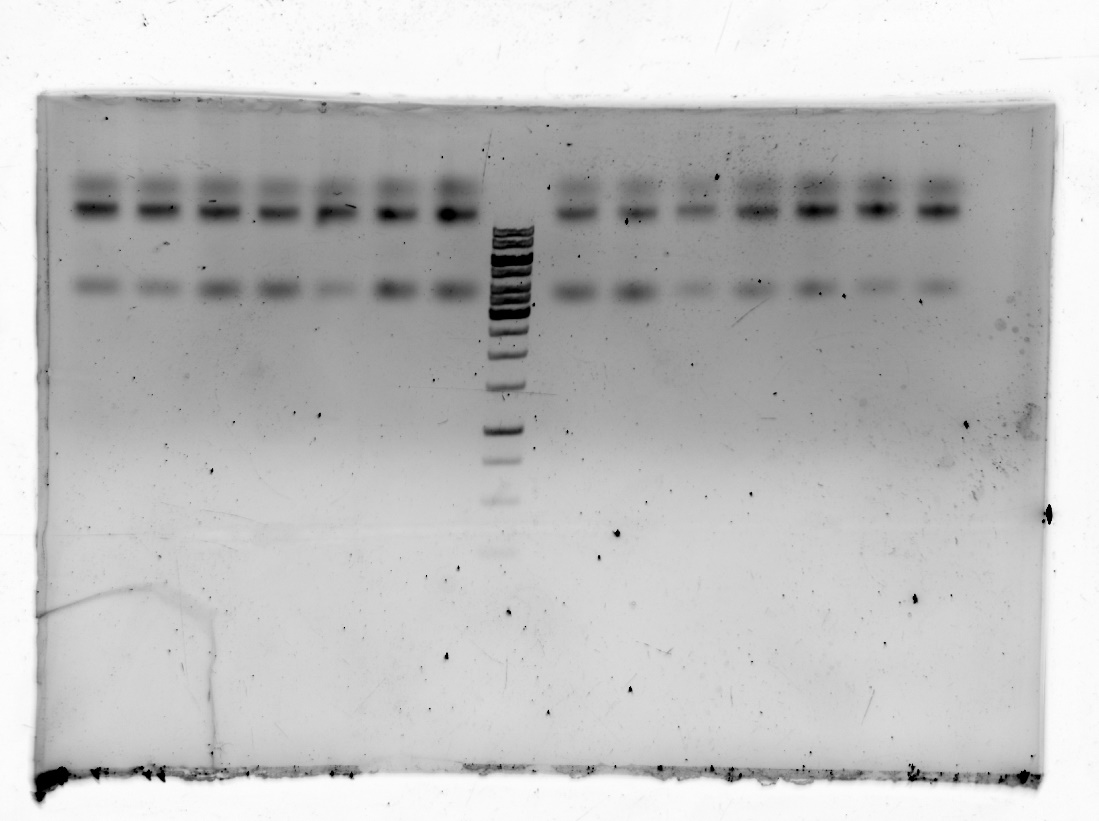
**

Samples 3-4

**Total GSK**


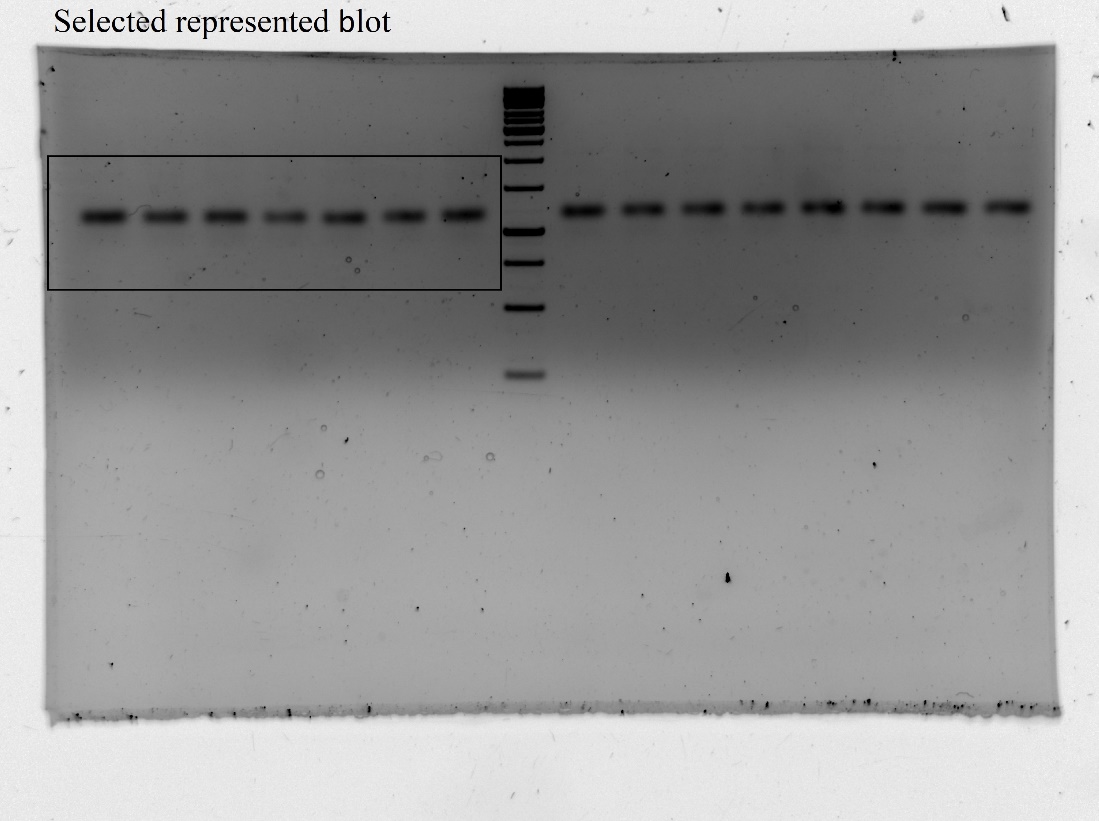


Samples 1-2


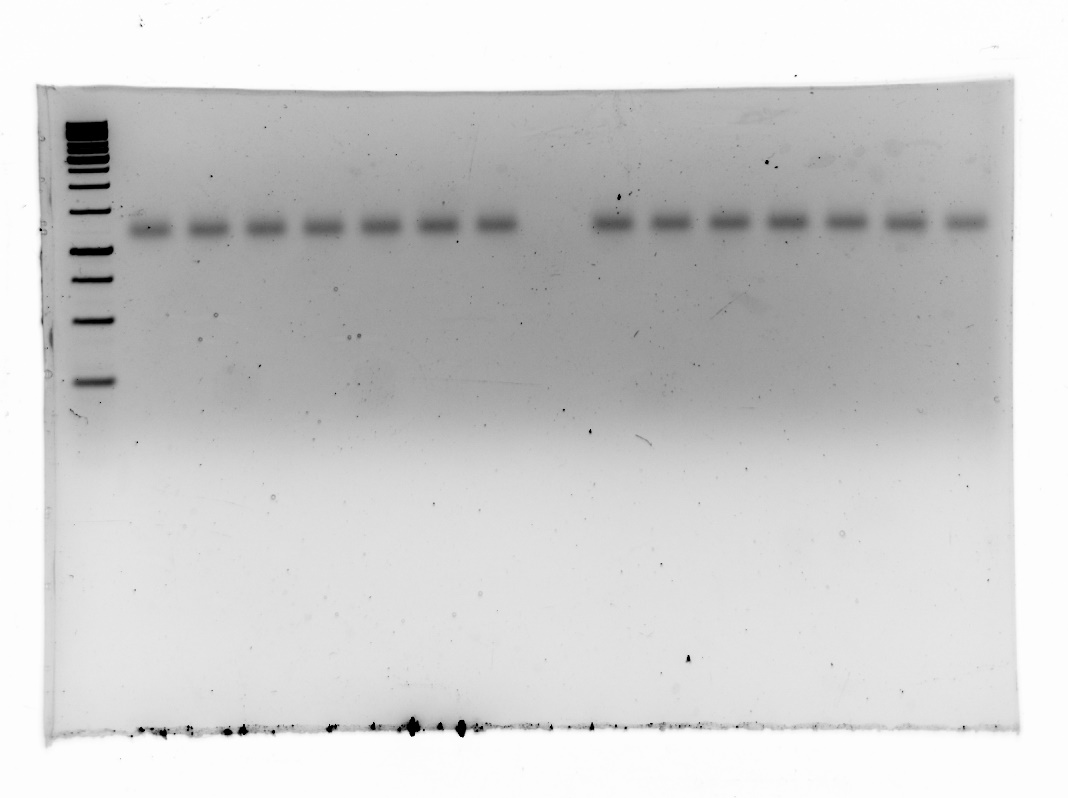


Samples 3-4

**Beta-catenin**

**
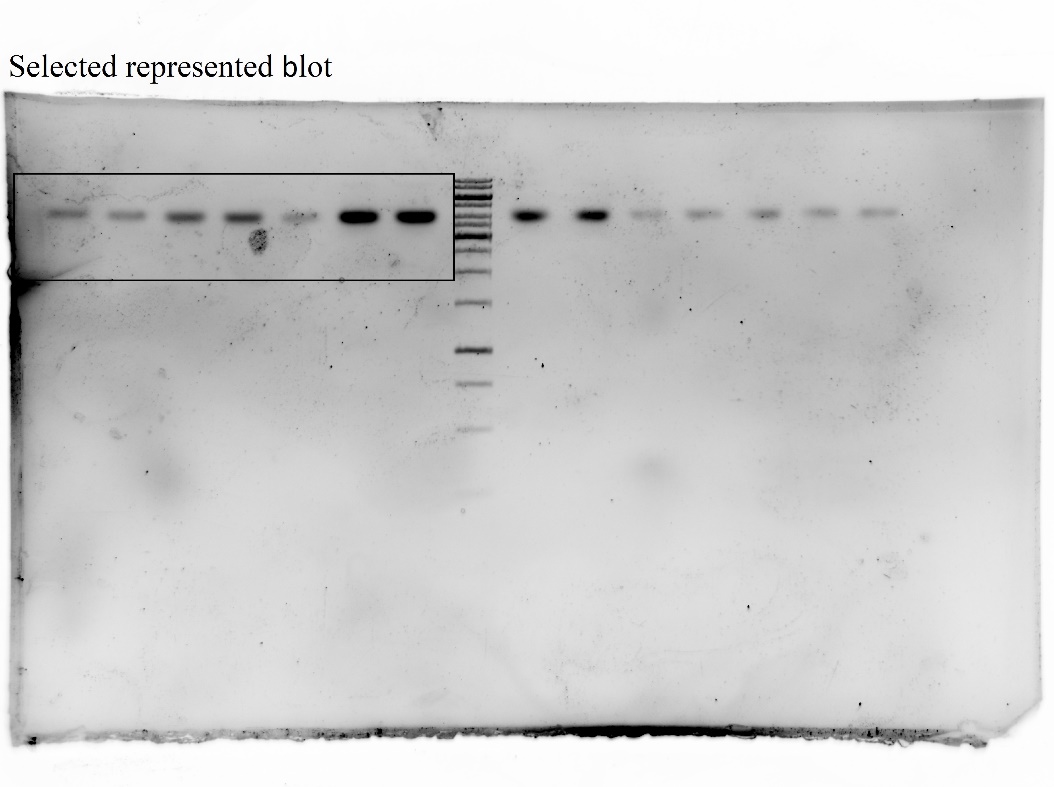
**

Samples 1-2

**
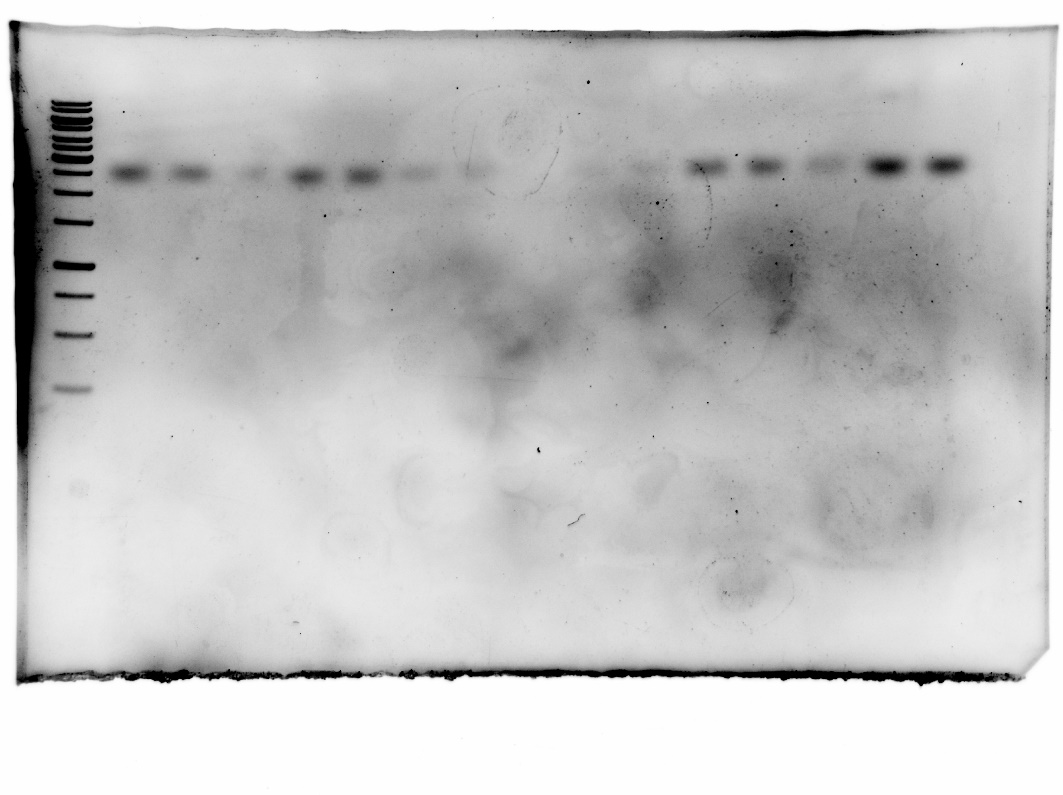
**

Samples 3-4

**Dicer**

**
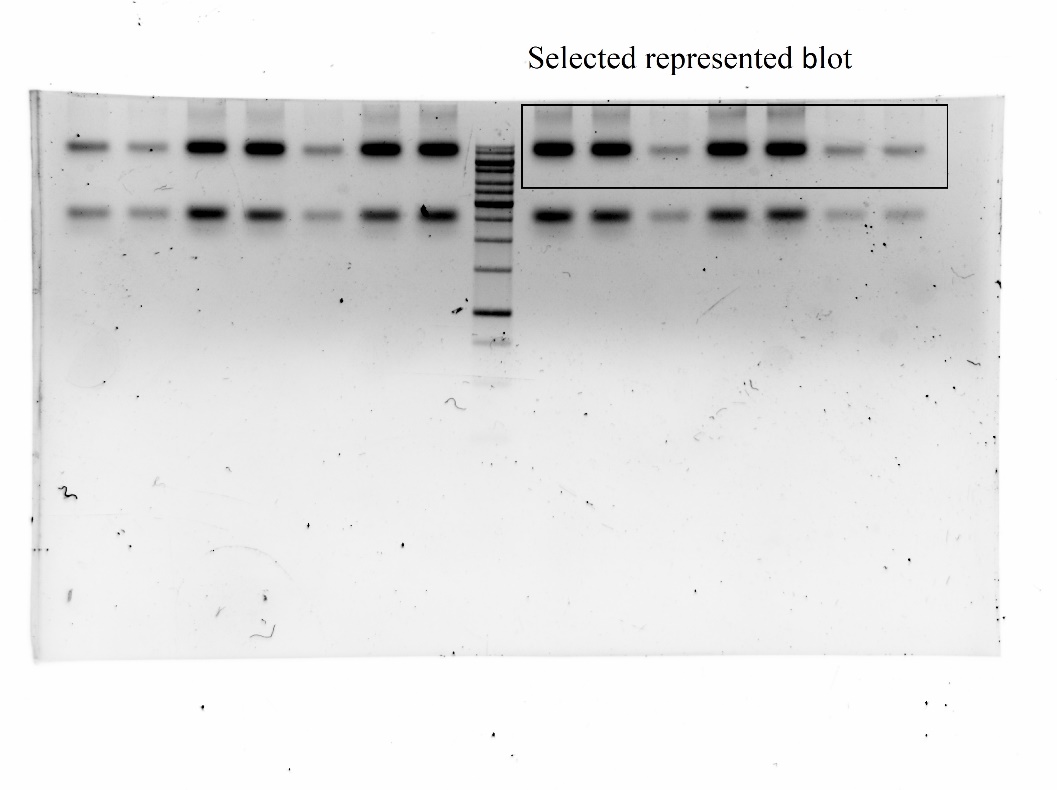
**

Samples 1-2

**
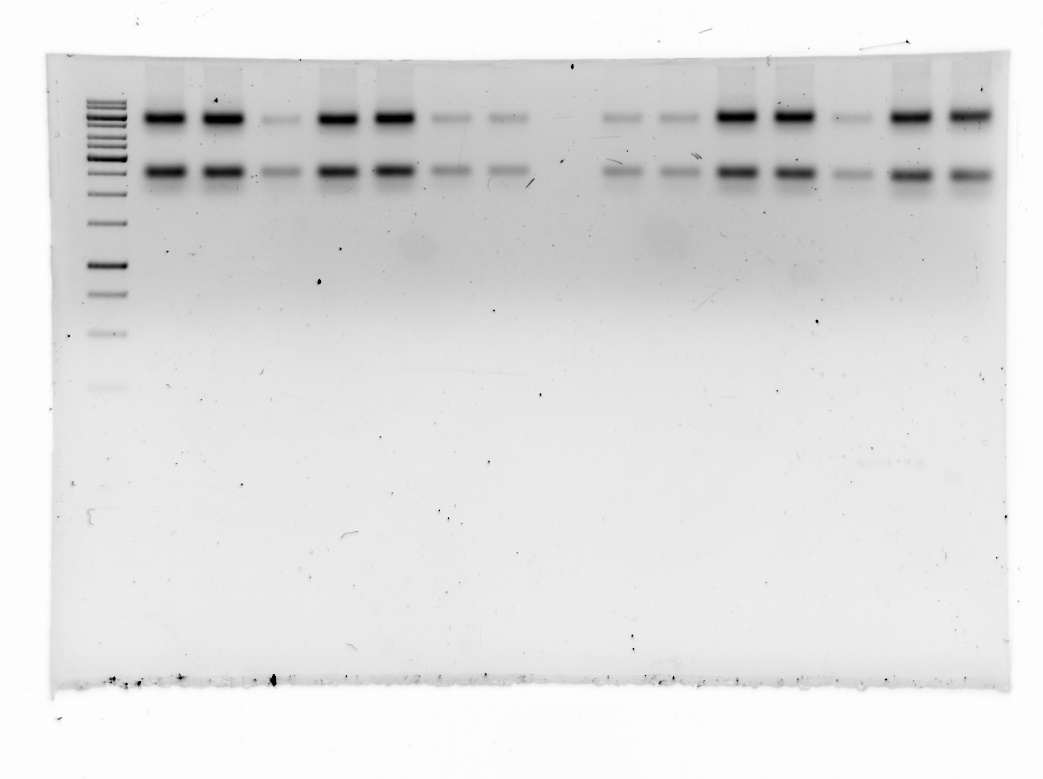
**

Samples 3-4
